# Meiosis-Specific Loading of the Centromere-Specific Histone CENH3 in *Arabidopsis thaliana*

**DOI:** 10.1371/journal.pgen.1002121

**Published:** 2011-06-09

**Authors:** Maruthachalam Ravi, Fukashi Shibata, Joseph S. Ramahi, Kiyotaka Nagaki, Changbin Chen, Minoru Murata, Simon W. L. Chan

**Affiliations:** 1 Department of Plant Biology, University of California Davis, Davis, California, United States of America; 2 Institute of Plant Science and Resources, Okayama University, Kurashiki, Japan; 3 Department of Horticultural Science, University of Minnesota, St. Paul, Minnesota, United States of America; The University of North Carolina at Chapel Hill, United States of America

## Abstract

Centromere behavior is specialized in meiosis I, so that sister chromatids of homologous chromosomes are pulled toward the same side of the spindle (through kinetochore mono-orientation) and chromosome number is reduced. Factors required for mono-orientation have been identified in yeast. However, comparatively little is known about how meiotic centromere behavior is specialized in animals and plants that typically have large tandem repeat centromeres. Kinetochores are nucleated by the centromere-specific histone CENH3. Unlike conventional histone H3s, CENH3 is rapidly evolving, particularly in its N-terminal tail domain. Here we describe chimeric variants of CENH3 with alterations in the N-terminal tail that are specifically defective in meiosis. *Arabidopsis thaliana cenh3* mutants expressing a GFP-tagged chimeric protein containing the H3 N-terminal tail and the CENH3 C-terminus (termed GFP-tailswap) are sterile because of random meiotic chromosome segregation. These defects result from the specific depletion of GFP-tailswap protein from meiotic kinetochores, which contrasts with its normal localization in mitotic cells. Loss of the GFP-tailswap CENH3 variant in meiosis affects recruitment of the essential kinetochore protein MIS12. Our findings suggest that CENH3 loading dynamics might be regulated differently in mitosis and meiosis. As further support for our hypothesis, we show that GFP-tailswap protein is recruited back to centromeres in a subset of pollen grains in *GFP-tailswap* once they resume haploid mitosis. Meiotic recruitment of the GFP-tailswap CENH3 variant is not restored by removal of the meiosis-specific cohesin subunit REC8. Our results reveal the existence of a specialized loading pathway for CENH3 during meiosis that is likely to involve the hypervariable N-terminal tail. Meiosis-specific CENH3 dynamics may play a role in modulating meiotic centromere behavior.

## Author Summary

There are two types of cell division in eukaryotes. Mitosis produces cells with identical copies of the genome, while meiosis produces gametes with half the number of chromosomes found in the parent cell. Faithful genome inheritance is controlled by centromeres, chromosomal structures that allow duplicated chromosomes to be pulled apart correctly during cell division. Centromeres are differentially configured during meiosis (relative to mitosis) so chromosome number can be reduced by half. Centromeres are built upon a specialized DNA packing protein, CENH3. Here we describe altered forms of CENH3 that are loaded correctly during mitosis but are severely depleted from centromeres in meiotic cells. As CENH3 is essential for chromosome inheritance, plants expressing these versions of the protein are sterile because they produce very few viable gametes. Differential loading of CENH3 during meiosis may play a role in modulating chromosome inheritance to form haploid gametes.

## Introduction

Centromeres are loci that direct faithful segregation of chromosomes during eukaryote cell division. They provide a platform for the assembly of kinetochores, structures that bind to spindle microtubules and coordinate chromosome movement. Centromere behavior must be regulated differently in mitosis and meiosis [1]. In mitosis, centromeres from sister chromatids face in opposite directions (bi-orientation), allowing the spindle to pull the replicated sisters apart at anaphase. In meiosis I, sister centromeres face in the same direction (mono-orientation). This allows sister chromatids to move to the same side of the spindle in anaphase I, when homologous chromosomes are segregated apart. Chromosome segregation errors in meiosis I are a primary cause of spontaneous abortion and birth defects, highlighting the importance of studying meiotic centromere behavior [1].

The mechanism of mono-orientation has been illuminated by yeast studies. In *Schizosaccharomyces pombe*, the meiosis-specific cohesin subunit Rec8 fuses sister kinetochores together in a geometry that favors attachment to microtubules from the same side of the spindle [2]. This appears to be a conserved mechanism, because *rec8* mutants in the plant *Arabidopsis thaliana* also show bi-oriented sister kinetochores in meiosis I [3]. Furthermore, fused sister kinetochores have been observed in maize meiosis I [4]. Other proteins required for mono-orientation in yeast, such as *S. pombe* Moa1p or the monopolin complex of *Saccharomyces cerevisiae*, are not found in animals or in plants [5], [6]. These proteins may evolve rapidly. However it is also possible that the mechanism of bi-orientation has different features between yeast kinetochores that are nucleated by small DNA sequences and animal and plant kinetochores that assemble on megabase-scale tandem repeat arrays [7].

Centromere function requires the centromere specific histone H3 variant (CENH3, called CENP-A in human), which replaces histone H3 in centromeric nucleosomes and recruits many essential kinetochore proteins [8]. Unlike conventional histones, which are extremely well conserved, CENH3s are fast evolving [9]. Genetic experiments in *A. thaliana* and in *S. cerevisiae*, as well as localization studies in *Drosophila melanogaster* have shown that evolutionarily divergent CENH3s cannot substitute for one another (although gene silencing experiments in human cells suggest greater promiscuity) [10]–[13]. The N-terminal tail domain of CENH3s is even more hypervariable than the C-terminal histone-fold domain, and shares almost no similarity between plant species such as *A. thaliana* and maize (*Zea mays*), let alone between plants and other eukaryotes [9], [13].

As the histone-fold domain of CENH3 is sufficient for kinetochore localization, the role of the tail domain is enigmatic. We have shown that a CENH3 protein lacking the tail is targeted to kinetochores, but fails to complement an *A. thaliana cenh3* null mutant [13]. However, replacing the CENH3 tail with the tail of conventional H3.3 in a GFP-tagged protein gives rise to viable but sterile plants (*cenh3* plants complemented with this transgene are referred to as *GFP-tailswap*) [14]. This unexpected result suggested that the CENH3 tail might have a specific function in meiosis, even though CENH3 is required for both mitotic and meiotic kinetochore functions. Most studies of CENH3 dynamics and function in eukaryotes with tandem repeat centromeres are limited to mitosis, since the knockout of CENH3 is zygotic lethal. The viability and subsequent sterility of *cenh3 GFP-tailswap* plants enabled us to investigate the meiosis specific role of CENH3 in *A. thaliana*.

Here we present a detailed analysis of the meiotic phenotype of *A. thaliana* plants expressing GFP-tagged CENH3 variants with alterations in the CENH3 tail domain. Sterility in these plants was caused by random chromosome segregation, with the first defects appearing at the onset of metaphase I (the stage at which centromere behavior is expected to differ between mitosis and meiosis). Chromosome segregation defects in meiosis were explained by severe depletion of the GFP-tailswap CENH3 variant at meiotic kinetochores (the same protein is loaded normally in mitosis). Depletion of CENH3 at centromeres also compromised the recruitment of the essential kinetochore protein Mis12. Our results thus reveal that centromeres have a meiosis-specific assembly mechanism which involves the CENH3 tail domain. This previously unsuspected pathway may play a role in modulating kinetochore dynamics to ensure differential centromere behaviour during meiosis.

## Results

### Viable but sterile *cenh3* variants suggest that CENH3 function differs between mitosis and meiosis

**Figure 1:**
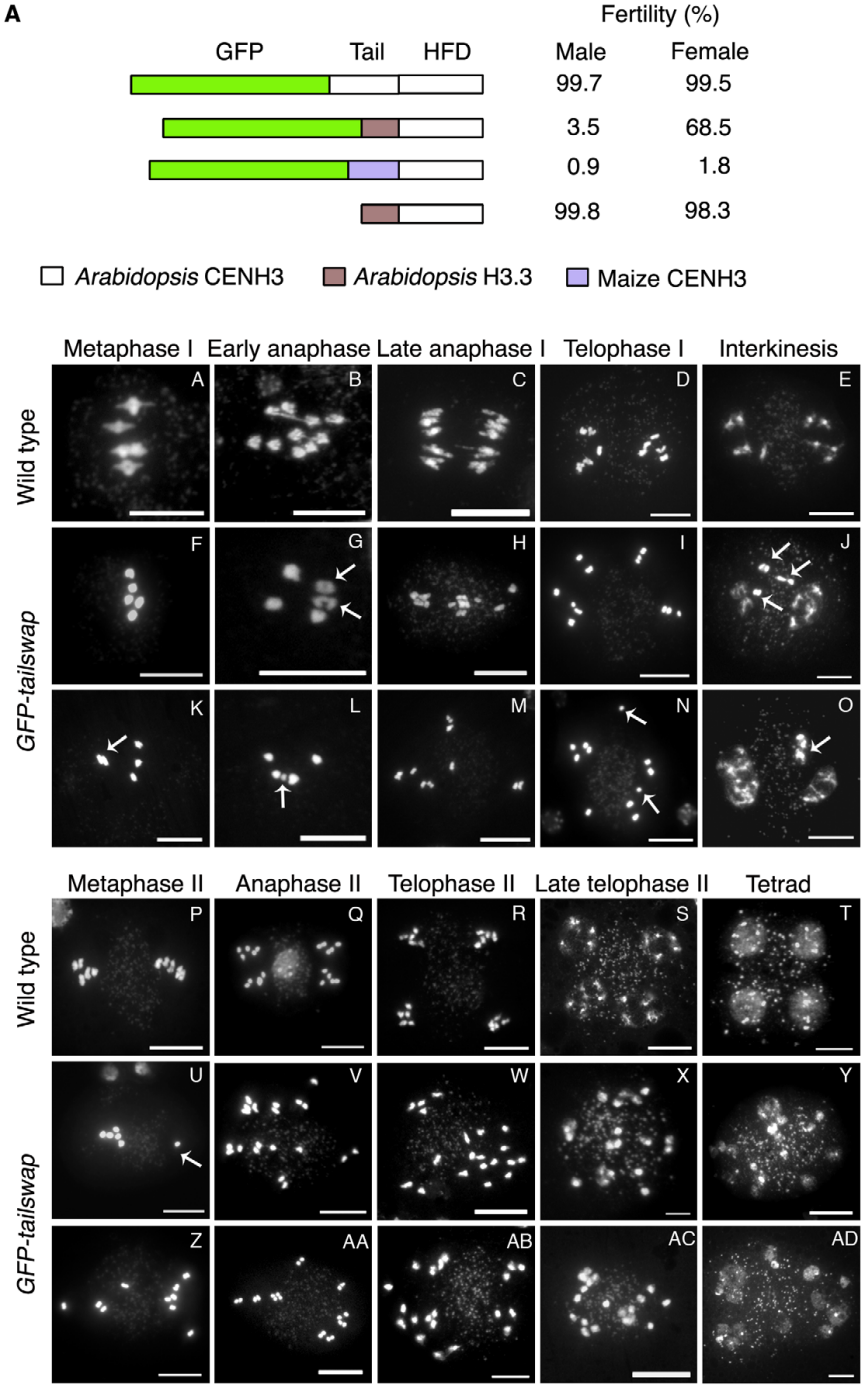
Altering the CENH3 N-terminal tail domain leads to defects in meiotic chromosome segregation. a) CENH3 transgenes tested for fertility in a *cenh3-1* homozygous mutant background. The male fertility was examined by Alexander staining. Viable pollen stains pink/red. Female fertility was judged by differential intereference contrast (DIC) microscopy of embryo sacs from at least 100 cleared mature ovules per genotype (Figure S1A). Single cell arrested ovules and ovules without an embryo sac (Figure S1B) were counted as non-viable, and ovules with 7–8 celled embryo sacs (Figure S1B) were counted as viable. Viable ovules may be haploid or aneuploid. b) Male meiotic chromosome spreads from wild type and *GFP-tailswap* plants. Metaphase I bivalents in the mutant are oval/round in shape, lacking the rhombus shape that indicates tension in wild type (compare A and F). Some metaphase I cells showed chromosomes that failed to congress to the spindle midzone (arrowed in K). Chromosome segregation at anaphase I is random in *GFP-tailswap* (G to I, L to N). Asynchronous homolog separation was seen at anaphase I (arrowed in G), and premature sister chromatid separation was also seen in meiosis I (arrowed in N). Decondensation at interkinesis was frequently delayed, especially for lagging chromosomes near the spindle midzone (arrowed in J, O). Metaphase II cells in the mutant show random chromosome alignment (U, Z). U shows one univalent (arrowed) and four bivalents plus the remaining univalent on the other side of the cell. Anaphase II chromosome segregation is random (V–X, AA–AC). Tetrad equivalent stages in *GFP-tailswap* (Y, AD) show several small nuclei instead of the expected four uniform nuclei seen in wild type. Scale bars −1 µm.

We previously observed sterility in *GFP-tailswap* plants but not in plants expressing *GFP-CENH3*, suggesting that the N-terminal tail of CENH3 might have a specific role in plant reproduction [14]. The sterile phenotype of *GFP-tailswap* could result from the absence of the CENH3 tail, or from the presence of the H3.3 tail. To differentiate between these possibilities, we created a chimera in which the *A. thaliana* CENH3 tail was replaced with an unrelated CENH3 tail domain from maize (*Zea mays*), and transformed it into *cenh3-1* heterozygotes (Figure 1A). This GFP-maizetailswap protein was targeted to kinetochores and rescued the embryo-lethal phenotype of *cenh3-1*. In contrast, full-length maize CENH3 protein was targeted to *A. thaliana* kinetochores but failed to rescue *cenh3-1* embryo lethality [13]. Complemented *GFP-maizetailswap* plants showed the vegetative phenotype previously seen in *GFP-tailswap* plants but were even more sterile than *GFP-tailswap* plants (although one partially fertile *GFP-maizetailswap* plant was recovered) (Figure 1A) [13].

A GFP tag can have a deleterious effect on CENH3 function [15]. Self-pollinated *GFP-CENH3* plants are phenotypically indistinguishable from wild type and fully fertile, indicating that the GFP tag does not interfere with meiosis. We constructed an untagged tailswap transgene to test the role of the CENH3 N-terminus in a protein lacking an N-terminal GFP (Figure 1A). Interestingly, *cenh3-1* plants expressing a *tailswap* transgene without GFP were viable and fertile, indicating that the meiosis-specific role of the CENH3 tail domain is evident only when protein function is compromised by the presence of an N-terminal GFP tag.

Together, these results suggest that either the H3.3 tail or the maize CENH3 tail in the place of the native *Arabidopsis* CENH3 tail can severely compromise plant reproduction when combined with a GFP tag. This is especially interesting because the CENH3 tail is not required for centromere localization during mitosis, and is extremely fast-evolving [9], [16].

### Sterility in tailswap *cenh3* variants is caused by meiotic chromosome segregation defects

Sterility in *GFP-tailswap* plants could be caused by meiotic defects (during sporogenesis), or by later defects in post-meiotic cell divisions (gametogenesis). To investigate the cause of sterility, we analyzed the course of male meiosis in chromosome spreads from anthers. The major early events of meiotic homolog pairing and recombination appeared normal in *GFP-tailswap* (Figure S2). In prophase I, progressive condensation of chromosomes, homolog pairing, chiasmata formation and subsequent desynapsis of homologs (at diplotene) were similar in *GFP-tailswap* (n = 317) and wildtype meioses (Figure S2).

The first defects in *GFP-tailswap* were seen in metaphase I (Figure 1B). In most mutant cells (98/112), bivalent chromosomes congressed normally to the spindle midzone (Figure 1B, panel F). However, a few (14/112) showed alignment defects such as widely spaced metaphase plates, and unaligned bivalent chromosomes (Figure 1B, panel K). Chromosomes can congress to the metaphase plate in the absence of a centromere [17]. Such movements may be driven by chromosome arm-associated kinesins that are independent of the presence of a functional kinetochore [18]. Plant genomes do not contain chromokinesins, the animal proteins that perform this role, but functional counterparts may exist.

A striking defect was seen in the shape of chromosomes during the metaphase I to anaphase I transition. In wild-type meiosis, bivalent chromosomes at this stage assume a rhombus- or linear-shaped configuration caused by tension between spindle microtubules pulling on the kinetochore and chiasmata that hold homologous chromosomes together (Figure 1B, panel A). In *GFP-tailswap* meiocytes, wild-type metaphase configurations were rarely observed.

Instead, bivalents were oval and irregularly shaped, resembling prometaphase I stage meiocytes (n = 79) (Figure 1B, panels F and K). Prometaphase stage chromosomes are rarely seen in wildtype meiotic spreads (in a total of 392 prophase stage meiocytes, only 4 of this type were observed) because this stage has only a short duration before the onset of metaphase I. Our observations suggest that meiocytes in *GFP-tailswap* stall at the prometaphase stage and proceed directly to anaphase I without going through a typical metaphase-anaphase transition stage at which tension is exerted by the spindle.

During anaphase I, *GFP-tailswap* bivalents frequently segregated both homologs to the same side of the spindle, in addition to the normal behavior of resolving homologs and segregating them to opposite poles (Figure 1B, panels G and L). In wild type, pulling forces from the meiosis I spindle help to resolve homologs at anaphase. In *GFP-tailswap*, homolog separation was more asynchronous than in wild type, yielding cells that contained a mixture of bivalents and univalents (Figure 1B, panels G, H, L and M). The bivalents then separated their homologs at a later stage of anaphase I (Figure 1B, panel M). Some cells contained lagging chromosomes at the spindle midzone, supporting the idea that attachment to the meiotic spindle is impaired (Figure 1B, panels J and O). In rare instances (4/103), sister chromatids separated prematurely during meiosis I (in contrast to normal separation at anaphase II) (Figure 1B, panel N). As a result of random chromosome segregation, meiosis I in *GFP-tailswap* yielded predominantly unbalanced dyads with 6-4, 7-3 configurations e.t.c. instead of the 5-5 segregation that is universally seen in wild type (reductional segregation to give 5-5 dyads was seen in 6% of mutant cells) (Figure 1B, panels J and O).

At interkinesis, an intermediate stage between meiosis I and II, wild-type chromosomes decondense and recondense. In *GFP-tailswap*, decondensation and recondensation were delayed in some chromosomes (especially those located at or near the midzone) (Figure 1B, panels J and O). Instead of regrouping and aligning on the metaphase plate during meiosis II, *GFP-tailswap* chromosomes remained scattered throughout the meiocyte in a manner similar to anaphase I chromosomes in the mutant (Figure 1B, panel Z). Anaphase II in wild type *A. thaliana* separates sister chromatids to form four groups of 5 chromosomes, each of which contains one haploid genome. In *GFP-tailswap*, sister chromatids separated while chromosomes were scattered, showing that anaphase II begins without correct chromosome alignment on the metaphase plate (Figure 1B, panels V and AA). Chromosome decondensation also occurred at dispersed locations throughout the cell (Figure 1B, panels X and AC). Instead of the wild type tetrad containing four haploid nuclei, *GFP-tailswap* meiocytes after meiosis II are polyads containing many small nuclei (in *A. thaliana*, cytokinesis occurs after the tetrad is formed) (Figure 1B, panels Y and AD). Analysis of male meiosis in *GFP-maizetailswap* plants revealed meiotic chromosome segregation defects similar to those in *GFP-tailswap* plants (Figure S3). Based on the chromosome segregation phenotypes described above, it is clear that sterility in *GFP-tailswap* and *GFP-maizetailswap* is caused by a severe meiosis-specific defect in centromere function.

### Centromere function is required to compact segregating chromosomes into a single nucleus in microspores

**Figure 2:**
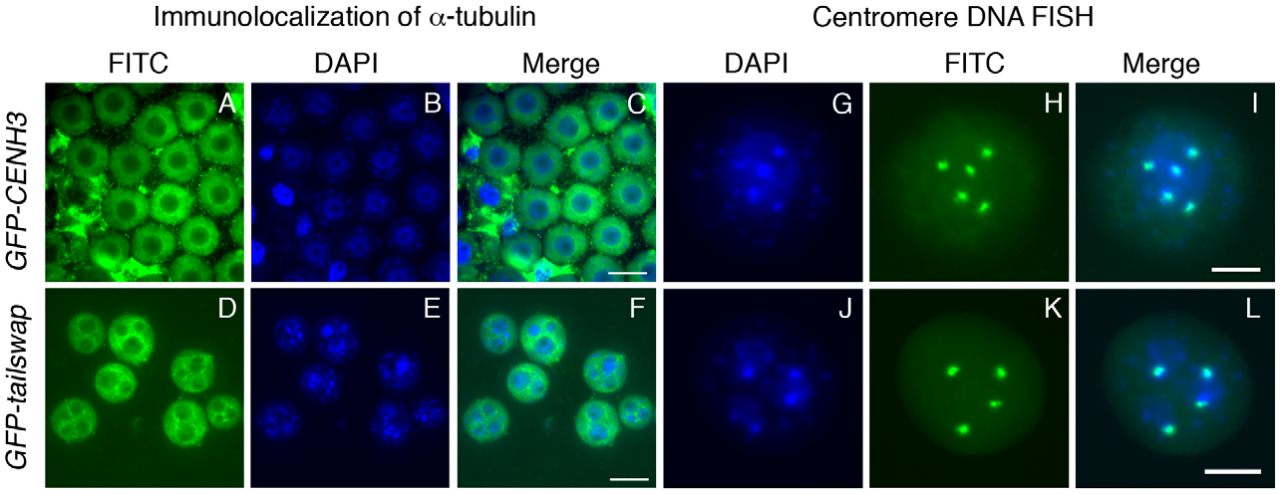
Lack of centromere function in meiosis causes micronuclei to form in *GFP-tailswap* pollen. Immunolocalization of alpha-tubulin outlines the nuclear envelope in microspores of *GFP-CENH3* and *GFP-tailswap* pollen (A–F). *GFP-tailswap* pollen contains multiple micronuclei. Centromere DNA FISH shows that micronuclei contain 1–2 chromosomes each, as opposed to 5 chromosomes in a normal *A. thaliana* haploid pollen genome (G–L). The pollen grain shown in J–L has three micronuclei. Two contain one chromosome each, while the third contains two chromosomes.

Analysis of microspores (pollen grains) from *GFP-tailswap* plants revealed that each contained 1–8 nuclei instead of the single nucleus that is always seen in wild type (Figure 2A–2F). These micronuclei varied in size, suggesting that they contained different numbers of chromosomes. Fluorescence in situ hybridization (FISH) using a probe that recognizes the 180 bp centromere tandem repeats confirmed that each micronucleus contained from 1–4 chromosomes (Figure 2J–2L). This observation suggests that randomly scattered chromosomes that lie in close proximity reassemble their own nuclear envelope at the end of telophase II, resulting in multiple micronuclei within each microspore. A similar defect has been reported in *A. thaliana* separase (*esp*) mutants defective in the enzyme that releases sister chromatid cohesion [19]. In mammalian somatic cells, micronuclei formation is triggered by the depletion of factors required for chromosome segregation [20]–[22]. Formation of micronuclei might be a general feature of perturbations that drastically affect chromosome movement in mitosis or meiosis.

Viable male and female gametes in *GFP-tailswap* are expected to contain a single nucleus with a haploid genome of five chromosomes, because most (95%) of the viable progeny from self fertilized *GFP-tailswap* are diploid [14], [13]. This can be contrasted with several plant meiotic mutants which show random chromosome segregation during meiosis [23]–[25]. After meiosis II, these mutants often contain more than the normal four nuclei within a single meiocyte. However, the microspores resulting from such meiocytes usually contain a single nucleus, in contrast to the multinucleate microspores of separase mutants and *GFP-tailswap*. In *A. thaliana* mutants with general meiotic defects (for example, *ask1* and *spo11*), a high fraction of viable gametes are aneuploid [24], [25]. When such plants are self-fertilized, a high fraction of viable progeny are aneuploid. Formation of micronuclei in the microspore selects against otherwise viable aneuploid pollen in *GFP-tailswap*, so >90% of progeny obtained by self-fertilization are diploid, despite random chromosome segregation in meiosis. We conclude that functional centromeres are required to package segregating chromosomes into a single pollen nucleus, and are thus necessary for accurate transmission of a haploid genome after meiosis.

### Chromosomes in *GFP-tailswap* meiosis appear to lack tension from the spindle and show abnormal alignment in metaphase I

**Figure 3:**
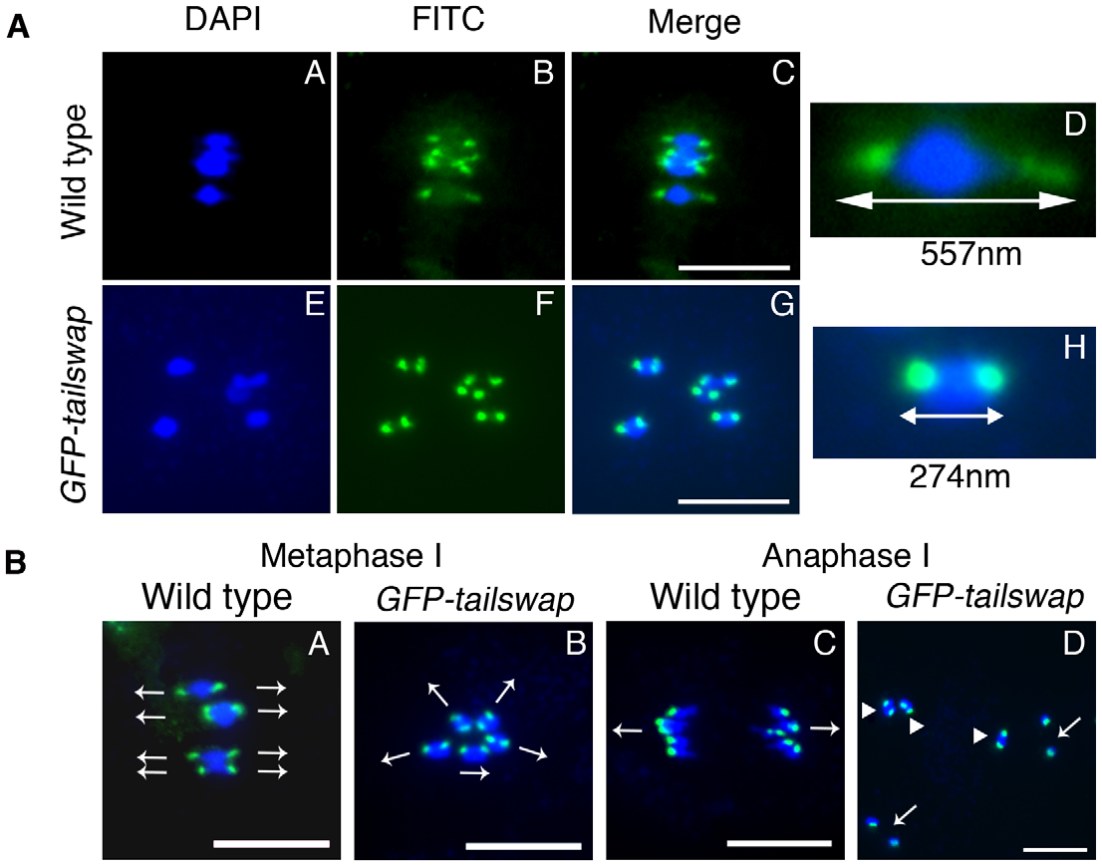
Reduced inter-kinetochore distance and meiotic spindle defects suggest lack of kinetochore function in *GFP-tailswap*. a) Centromere DNA FISH from metaphase I in wild type and in *GFP-tailswap*. Blue = DNA (DAPI), green = centromere DNA FISH (FITC). *GFP-tailswap* bivalents lack the centromere stretch exerted by the spindle in wild type, and have reduced inter-kinetochore distance. Representative bivalents are magnified in D and H. Scale bars −1 µm. b) Centromere DNA FISH shows random orientation of bivalent chromosomes in *GFP-tailswap* meiosis I. Blue = DNA (DAPI), green = centromere DNA FISH (FITC). Metaphase I chromosomes are frequently aligned at unusual angles in the mutant (B). Anaphase I chromosomes show random alignment and premature sister chromatid separation (D). Arrows in A and B show presumed orientation of sister centromeres. Arrows in D indicate separated univalents, while arrowheads show intact bivalents. Scale bars −1 µm.

A defect in kinetochore attachment to spindle microtubules in *GFP-tailswap* is suggested by the lack of apparent tension in metaphase I chromosomes and by random chromosome segregation (in the absence of pairing or recombination defects). The distance between opposing kinetochores (interkinetochore distance) during metaphase is a more precise measure of tension generated by the spindle during mitosis. To investigate interkinetochore distance in *GFP-tailswap* meiosis I, we used FISH with a centromere tandem repeat probe (Figure 3A). In meiotic chromosome spreads, the outer limit of centromere DNA staining indicates the likely position of the kinetochore. Wild type cells at the metaphase I to anaphase I transition showed centromere DNA foci whose outer edges were separated by a 405±68 nm distance (n = 15 bivalent chromosomes). Centromere DNA was clearly stretched out on either side of the non-hybridizing DNA representing chromosome arms (Figure 3A, panel D). In *GFP-tailswap* chromosomes at an equivalent stage, centromere DNA extremities were much closer to each other at 234±30 nm (n = 15 bivalent chromosomes) (Figure 3A, panel H). Furthermore, the centromere DNA stretch characteristic of wild-type chromosomes under tension was not seen in the mutant. We conclude that *GFP-tailswap* kinetochores may not be efficiently pulled by spindle microtubules.

FISH analysis of metaphase I meiocytes in *GFP-tailswap* also revealed abnormal alignment of centromeres with respect to the cell plate (Figure 3B). In wild-type meiosis I, centromeres from homolog pairs align in a direction perpendicular to the future division plane. In *GFP-tailswap* they aligned in multiple directions, presaging the random chromosome segregation that occurs at anaphase I. This data further supports the hypothesis that spindle microtubules fail to pull on kinetochores in *GFP-tailswap*, leading to a lack of tension and incorrect chromosome orientation during meiosis I.

### GFP-tailswap protein is loaded normally onto mitotic kinetochores but not onto meiotic kinetochores

**Figure 4:**
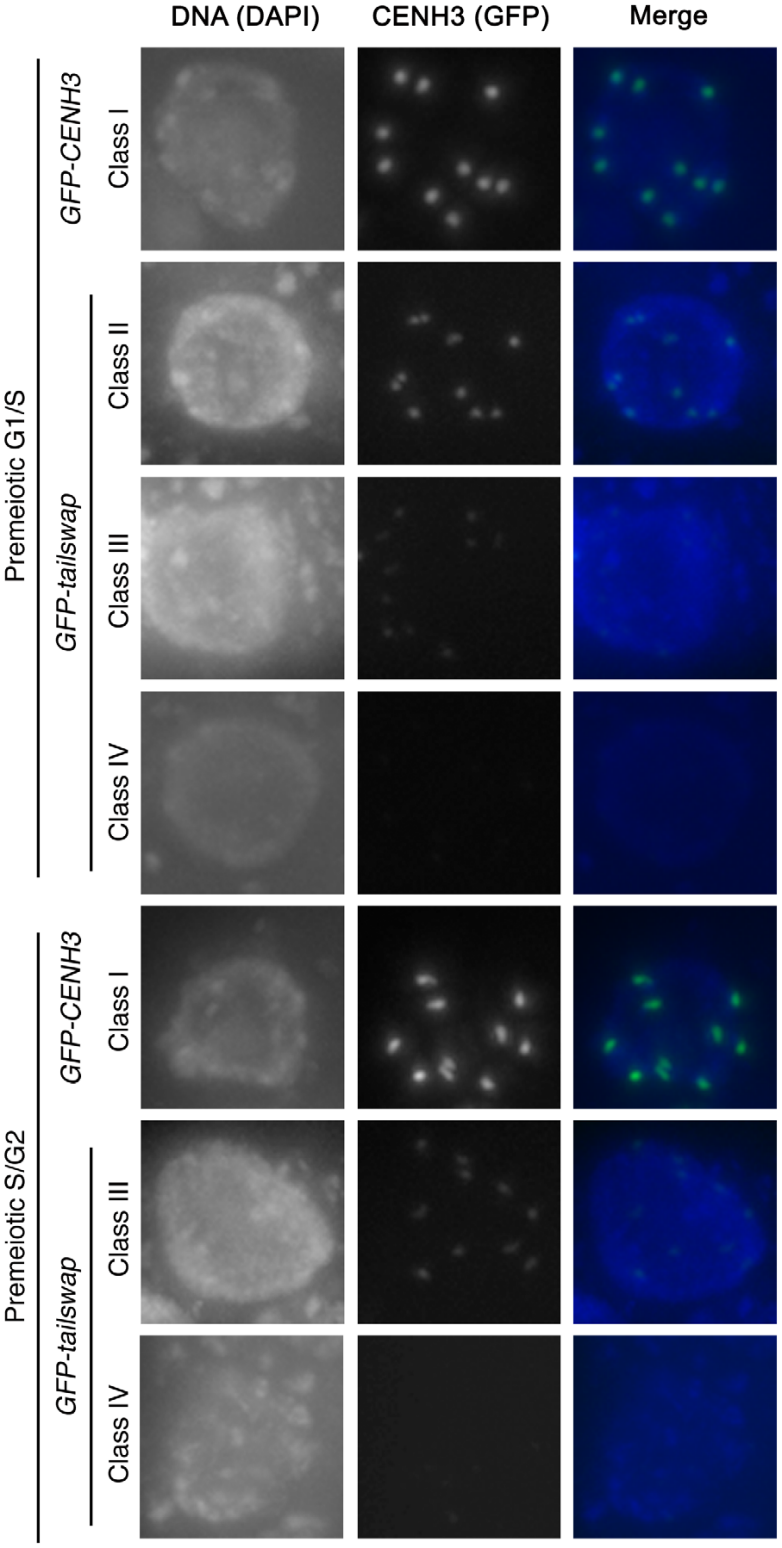
GFP-tailswap protein is depleted from kinetochores during pre-meiotic interphase. Meiocytes from anthers of *GFP-CENH3* and *GFP-tailswap* were imaged using identical exposure times. Flattened projections of several stacked images are shown. The GFP-CENH3 protein showed bright fluorescence at kinetochores in all meiocytes (class I). GFP-tailswap protein always showed reduced fluorescence relative to GFP-CENH3. Three classes of GFP fluorescence were observed in *GFP-tailswap*: faintly visible (class II, 7%), barely detectable (class III, 47%), and undetectable (class IV, 46%).

**Figure 5:**
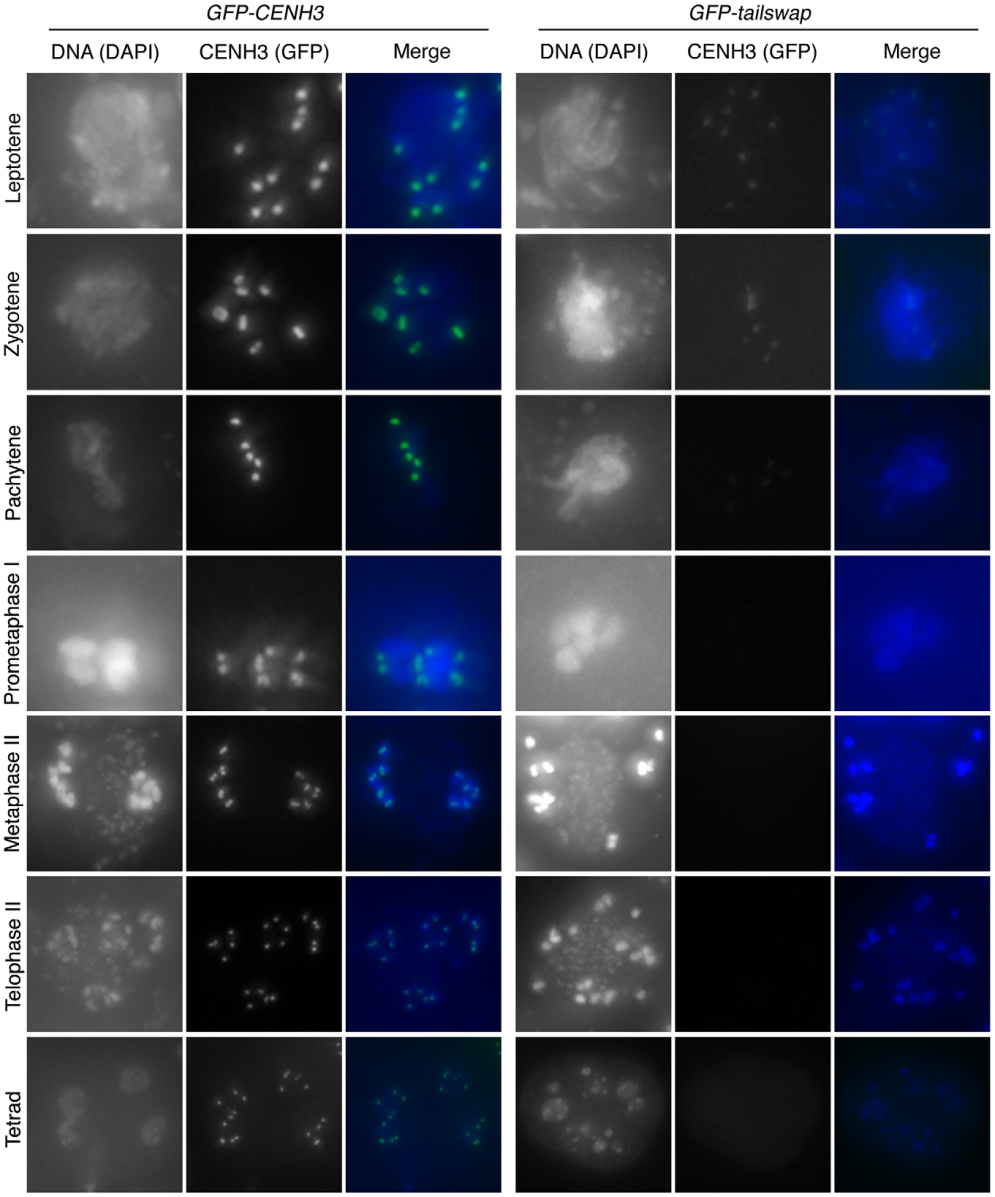
Depletion of GFP-tailswap protein from kinetochores continues progressively during meiosis. a) Dynamics of kinetochore GFP-CENH3 and GFP-tailswap proteins during meiosis were visualized in anthers. Flattened projections of several stacked images are shown. GFP-CENH3 is visible at uniform intensity at kinetochores throughout meiosis. GFP-tailswap is barely detectable or undetectable in leptotene and zygotene (comparable to class III and class IV in Figure 4). In pachytene and subsequent stages, we did not detect kinetochore GFP fluorescence in *GFP-tailswap* meiocytes.

As meiotic kinetochores appeared to be non-functional in *GFP-tailswap* plants, we investigated whether the GFP-tailswap variant of CENH3 was localized to meiotic kinetochores (Figure 4 and Figure 5). We have previously shown that GFP-tailswap protein is localized normally to mitotic kinetochores, and that mitosis is accurate in *GFP-tailswap* plants [13]. *A. thaliana* male meiocytes can be extruded as a cell conglomerate by gently squeezing anthers [26]. We imaged meiocytes from *GFP-CENH3* plants, and found that the GFP-tagged CENH3 protein was visualized at kinetochores in all stages of meiosis, as well as in haploid pollen grains. However in *GFP-tailswap* meiocytes, the protein was only faintly visualized at kinetochores during premeiotic and early prophase I stages (Figure 4 and Figure 5) and was not detected in later stages of meiosis I (starting from pachytene) and meiosis II. Depletion of GFP-tailswap from meiotic kinetochores contrasted with somatic cells from the same anther, which showed GFP fluorescence at mitotic kinetochores that appeared identical to wild-type (Figure S4).

**Figure 6:**
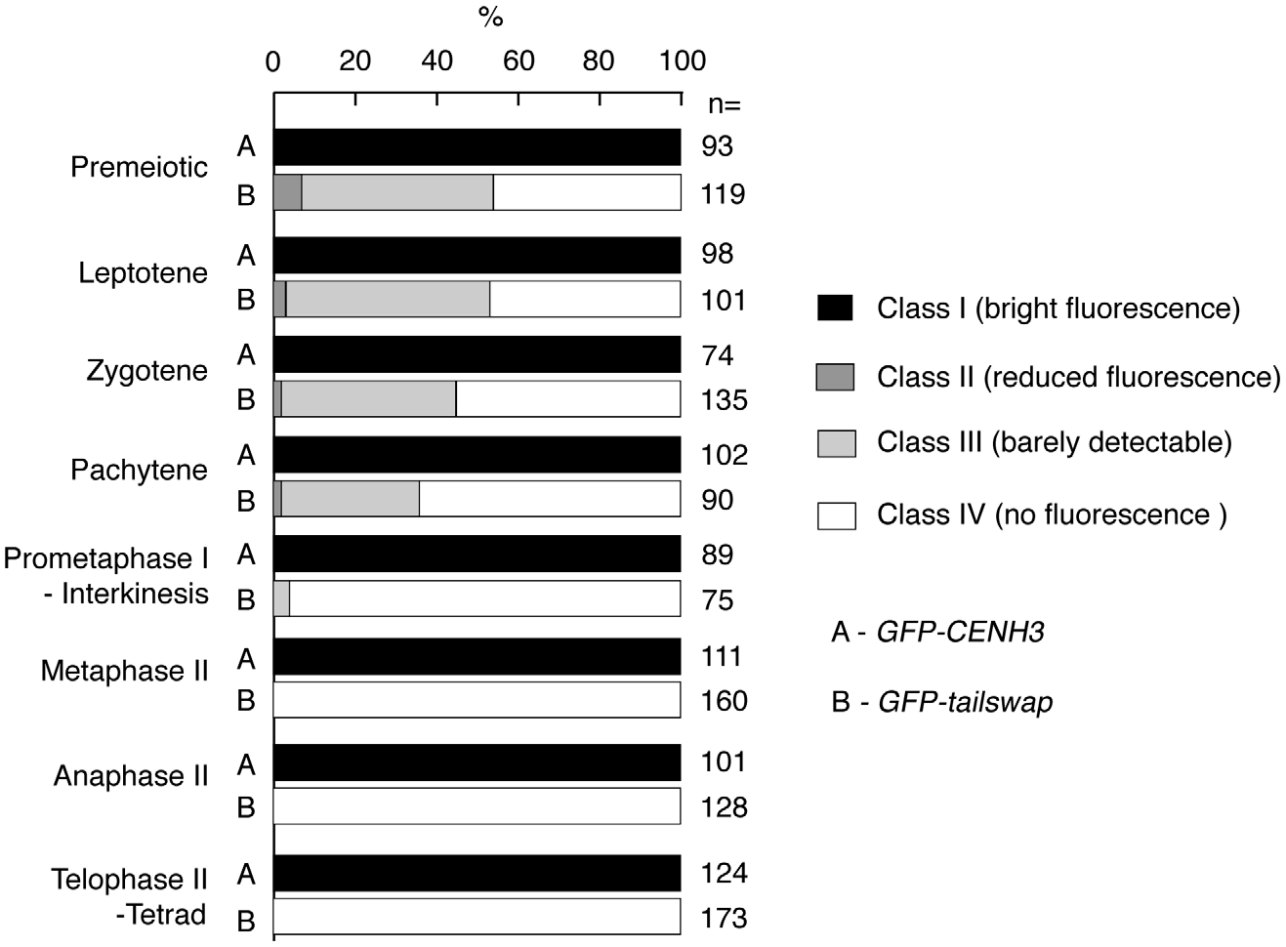
Percentage of *GFP-CENH3* and *GFP-tailswap* meiocytes showing particular classes of GFP fluorescence at kinetochores.

To further understand the dynamics of the GFP-tailswap protein during meiosis, we analyzed kinetochore GFP fluorescence at all meiotic stages from anther squashes (identification of these stages is described in the Materials and Methods). In premeiotic interphase of *GFP-CENH3* meiocytes, kinetochore GFP fluorescence was bright and uniform (Figure 4) (n = 93, 4 plants). *GFP-tailswap* meiocytes never showed bright kinetochore GFP fluorescence (Figure 4) (n = 119, 5 plants). Instead, we categorized them into three classes of reduced fluorescence, where GFP-CENH3 fluorescence is class I: 7% of meiocytes showed reduced kinetochore signal (class II), 47% had barely detectable fluorescence (class III), and the remaining 46% showed no GFP at kinetochores (class IV). This observation suggests that the GFP-tailswap protein is not replenished during premeiotic interphase, and that GFP-tailswap protein inherited from the somatic precursor cell is gradually removed from the centromere. Depletion of GFP-tailswap protein from meiocytes continued in subsequent stages of meiosis I (Figure 5 and Figure 6). Kinetochore GFP signal gradually disappeared during leptotene and zygotene stages of early prophase I (Figure 5). From late pachytene stage onwards until the completion of meiosis we could not detect GFP signal in any meiocytes (Figure 5 and Figure 6). We also used anti-GFP antibodies to immunolocalize GFP-CENH3 and GFP-tailswap proteins during meiosis, and found similar results (Figure S5). Residual GFP-tailswap protein may remain at kinetochores at a level below the detection limit. To verify that the GFP-tailswap mRNA was correctly spliced during meiosis, we extracted meiocytes from *GFP-CENH3* and *GFP-tailswap* anthers using a capillary-based method [26]. RT-PCR and subsequent sequencing of cDNA showed that the GFP-tailswap mRNA was identically spliced in somatic and meiotic cells (Figure S6). As CENH3 is essential, specific depletion of GFP-tailswap from meiotic kinetochores explains the chromosome missegregation that leads to sterility in *GFP-tailswap* plants.

The GFP-maizetailswap protein was also absent from meiotic kinetochores but present normally at mitotic kinetochores. Plants that co-express either *GFP-tailswap* or *GFP-maizetailswap* along with a wild-type endogenous *CENH3* gene were fully fertile. However, the GFP-tailswap and GFP-maize-tailswap proteins were poorly loaded onto meiotic kinetochores even in the presence of functional endogenous CENH3 (importantly, GFP-CENH3 loads normally in the presence of wild type CENH3) (Figure S7). Thus, the CENH3 tail domain appears to be required specifically for recruitment of the protein to meiotic kinetochores (when protein function is compromised by a GFP tag).

Kinetochore proteins that act only during meiosis have been described [1]. To our knowledge, this is the first example of an alteration in CENH3 that causes a meiosis-specific defect but allows for accurate mitosis. In *A. thaliana*, CENH3 is recruited to mitotic kinetochores after S phase, to replenish kinetochore CENH3 levels that were diluted by DNA replication [16]. If the GFP-tailswap and GFP-maizetailswap proteins were simply unable to replenish kinetochores after pre-meiotic DNA replication, we would expect to see half the GFP signal found at mitotic kinetochores in meiosis I cells. The fact that these proteins are greatly reduced at almost all meiotic kinetochores suggests that CENH3 chromatin is actively disassembled during meiosis in mutant plants. We believe that the *GFP-tailswap* and *GFP-maizetailswap* mutants reveal a meiosis-specific kinetochore assembly pathway whose existence was previously unsuspected.

### Depletion of the GFP-tailswap CENH3 variant from meiotic kinetochores causes loss of MIS12

**Figure 7:**
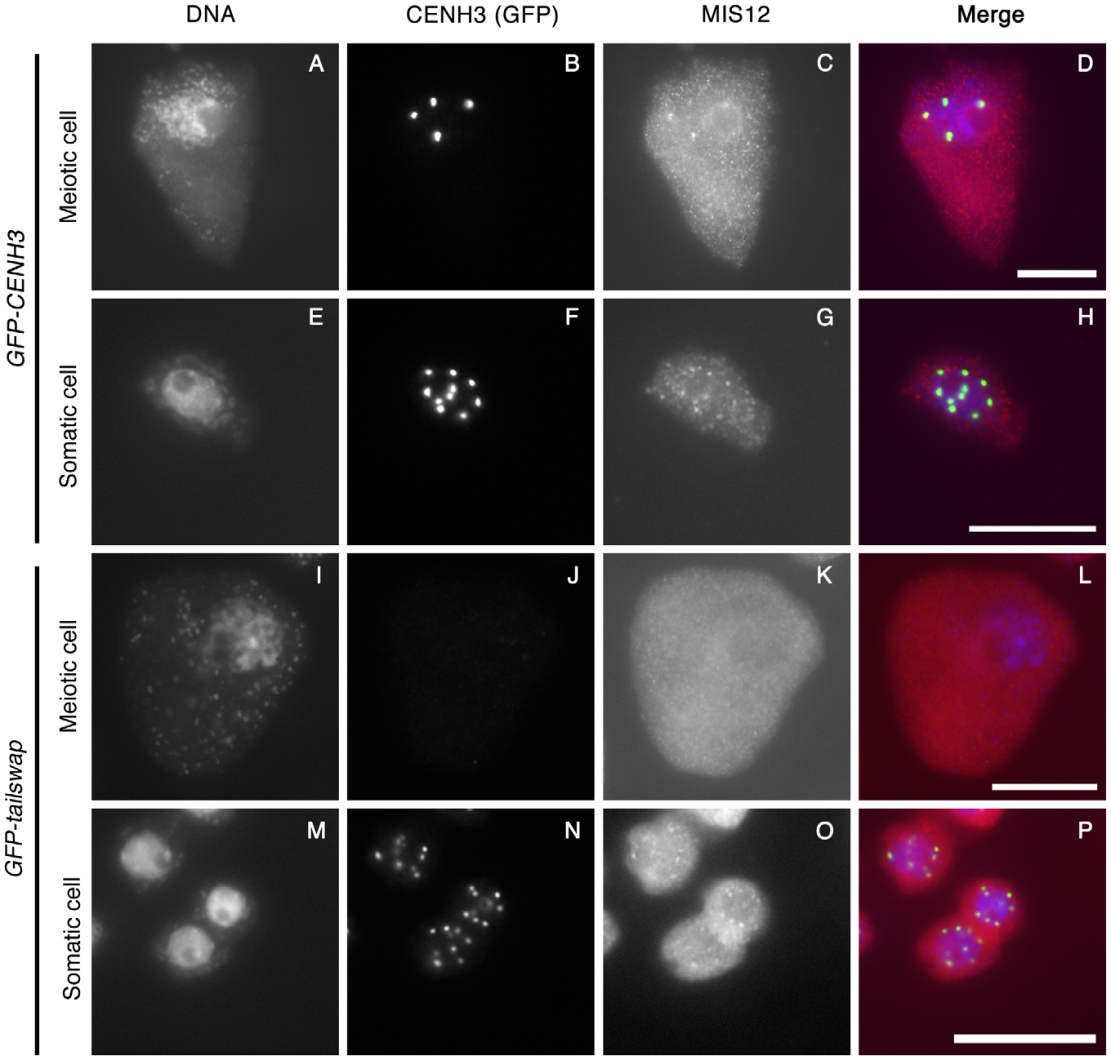
Depletion of GFP-tailswap protein from meiotic kinetochores causes removal of MIS12. GFP-CENH3, GFP-tailswap and MIS12 proteins were immunolocalized in anthers during the pachytene stage of meiosis with anti-GFP and anti-MIS12 antibodies. Somatic cells from the same anther are shown as a control. GFP-CENH3 and MIS12 were visualized at both meiotic and somatic kinetochores of *GFP-CENH3* plants. In *GFP-tailswap* plants, GFP-tailswap and MIS12 were both undetectable in *GFP-tailswap* meiotic kinetochores but can be seen in somatic kinetochores. Scale bars −5 µm.

CENH3 is required to recruit a large number of essential kinetochore proteins in other organisms. To further characterize the effects of the GFP-tailswap variant on kinetochore assembly, we performed immunostaining on *GFP-tailswap* and control *GFP-CENH3* anther squashes with antibodies raised against the *A. thaliana* kinetochore proteins CENP-C and MIS12 [27], [28]. CENP-C antibodies did not yield specific staining of kinetochores in meiocytes from either *GFP-CENH3* or *GFP-tailswap* plants. However, MIS12 staining was observed at kinetochores in *GFP-CENH3* meiocytes (n = 44), but not in *GFP-tailswap* meiocytes (n = 33) (Figure 7C and 7K). Somatic cells from both *GFP-CENH3* and *GFP-tailswap* plants showed kinetochore localization of MIS12 (Figure 7G and 7O). Although MIS12 may be recruited in a CENH3-independent way in human cells [20], our results show that loss of *A. thaliana* CENH3 in meiosis also depletes MIS12 from the kinetochore. As MIS12 is a component of the KMN network that connects kinetochores to spindle microtubules, we predict that this will compromise kinetochore-microtubule attachment [29]. Furthermore, MIS12 is important for mono-orientation during meiosis in maize [4]. In summary, severe depletion of the GFP-tailswap protein during meiosis and downstream effects on kinetochore assembly can explain the chromosome segregation defects observed in the mutant.

### Meiotic spindles are disordered in *GFP-tailswap*

**Figure 8:**
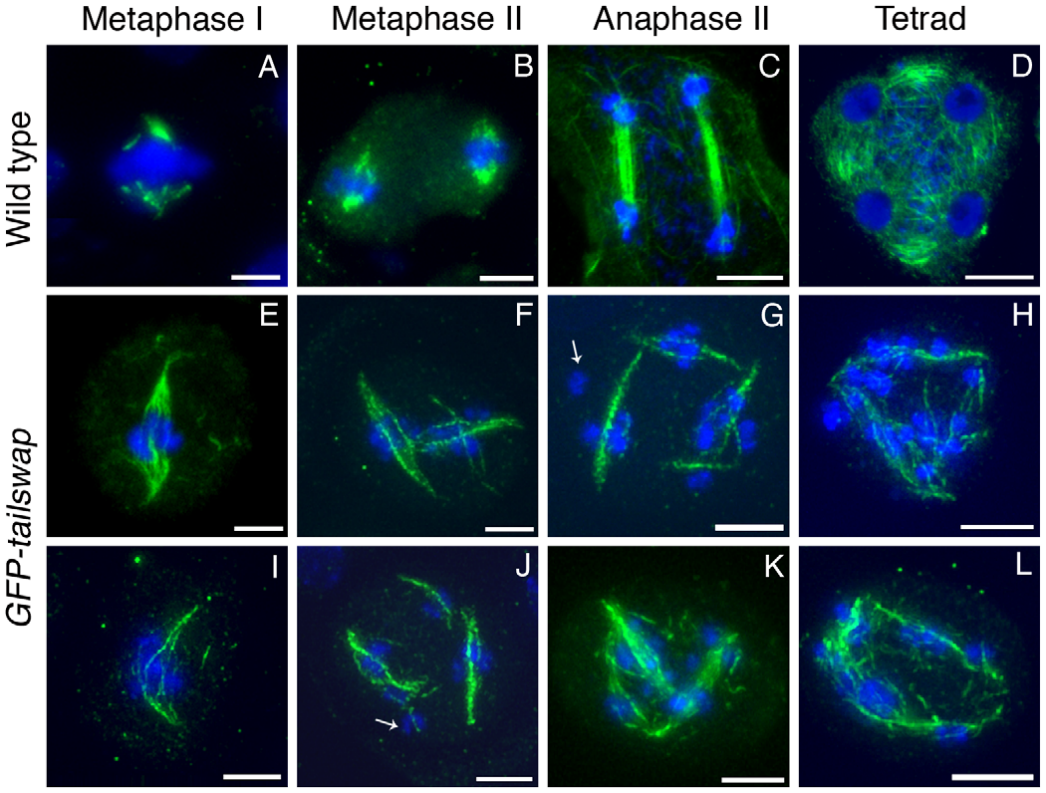
Immunolocalization of α-tubulin in wild type and *GFP-tailswap* meiocytes. Anther meiocytes from wild type and *GFP-tailswap* plants were stained with anti-tubulin antibodies. Metaphase I spindles are disorganized and longer in *GFP-tailswap*, and may contain fewer microtubules (E and I). Meiosis II cells in *GFP-tailswap* often contain more than two spindles (F–G, J–K). Spindle appearance and orientation are disordered, and may fail to include some chromosomes (arrowed in G). Tetrad equivalent cells (H, L) lack the radial microtubule system that surrounds the four haploid nuclei in wild type. Scale bars −5 µm.

Depletion or removal of CENH3 or other essential kinetochore proteins from the centromere results in compromised kinetochore function, which destabilizes the formation of a normal spindle [30], [31]. To gain insight into kinetochore-spindle microtubule interactions in *GFP-tailswap*, we visualized microtubules in meiocytes with anti-alpha-tubulin antibodies (Figure 8). A bipolar spindle is formed during metaphase I in mutants, but it was longer and more disorganized than the wild-type meiosis I spindle (Figure 8, panel E and I). Although we cannot conclude that kinetochores in *GFP-tailswap* are completely non-functional, our data is consistent with previous studies showing that kinetochores are not required to assemble a bipolar spindle in either mitosis or meiosis [32], [33].

It is possible that interactions between spindle microtubules and chromosome arm-binding kinesins can organize a spindle in the absence of fully functional kinetochores. In general, longer spindles are correlated with smaller kinetochores, and with abnormal chromosome movement [20]. This provides further evidence that kinetochores are functionally compromised in *GFP-tailswap* meiocytes.

Meiosis II spindles were even more disorganized in *GFP-tailswap* (Figure 8). Many meiocytes at this stage contained more than two spindles (multipolar spindles). Spindles were frequently perpendicular to each other or generally lacking the neat parallel appearance of spindles in wild-type meiosis II (Figure 8, panels F, G, J and K). These phenotypes may explain the inability of chromosomes to align on the metaphase II plate. Some kinetochores in *GFP-tailswap* meiocytes appeared to lack nearby spindle microtubules (Figure 8, panels G and J). However, it is difficult to conclude from our data that kinetochores fail to bind stably to spindle microtubules in the mutant, because the detection limit of tubulin staining in meiocytes is unknown. Furthermore, we cannot easily distinguish kinetochore microtubules from interpolar microtubules.

### GFP-tailswap recruitment to meiotic kinetochores is not restored by imposing mitosis-like chromosome behavior during meiosis I

If GFP-tailswap has a specific defect in recruitment to meiotic centromeres, can we suppress this phenotype by imposing a mitosis-like behavior on kinetochores in meiosis I? REC8 is a meiosis-specific cohesin subunit that functionally replaces its mitotic counterpart RAD21. REC8 is required to hold sister kinetochores together and force them to orient towards the same side of the spindle [3], [2]. In *S. pombe rec8* mutants, sister kinetochores in meiosis I show bipolar attachment to the spindle and segregate apart from each other, much as they do in mitosis [34]. The role of REC8 in mono-orientation is conserved in plants, as shown by *A. thaliana rec8 spo11* mutants (the *spo11* mutation is needed to prevent chromosome fragmentation, as *rec8* mutants cannot repair meiotic double-stranded breaks) [3]. To test whether CENH3 recruitment employs the mitotic loading pathway when REC8 is removed from meiotic kinetochores, we generated *rec8 spo11-1 cenh3-1* triple mutant plants carrying the *GFP-tailswap* transgene. If removing REC8 in *GFP-tailswap* mutants fully converted kinetochores from meiotic to mitotic behavior, we expected to see two experimental readouts. First, GFP-tailswap would be recruited to meiotic kinetochores in a manner similar to GFP-CENH3. Second, chromosomes in meiosis I would show a mitosis-like segregation pattern similar to the *rec8 spo11-1* mutant, because functional centromeres would be restored.

**Figure 9:**
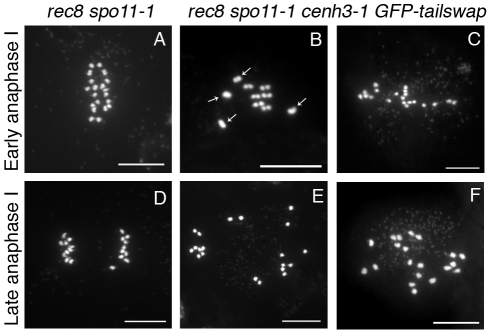
Removing the meiosis-specific cohesin REC8 does not restore meiotic kinetochore function in *GFP-tailswap*. Chromosome spreads from male meiosis in *rec8 spo11-1* and *rec8 spo11-1 cenh3-1 GFP-tailswap*. Anaphase I in *rec8 spo11-1* shows orderly separation of sister chromatids that is similar to mitosis, because the *rec8* mutation converts kinetochores to a mitosis-like behavior (A, D). Anaphase I in *rec8 spo11-1 cenh3-1 GFP-tailswap* shows random segregation of univalent chromosomes (B, C, E, F). This is consistent with the observation that removing REC8 does not restore loading of the GFP-CENH3 protein (Figure S6).

GFP-tailswap protein was not loaded onto meiotic kinetochores in *rec8 spo11-1 cenh3-1 GFP-tailswap* plants, showing that REC8 removal does not restore the mitotic CENH3 loading pathway during meiosis (Figure S8). In *rec8 spo11-1* mutants, chromosomes remain as univalents during prophase I and separate their sister chromatids at anaphase I as they do in mitosis (10-10 segregation instead of 5-5 segregation) (Figure 9 and Figure S8) [3]. By contrast, meiosis I in *rec8 spo11-1 cenh3-1 GFP-tailswap* plants showed random segregation of the unpaired univalent chromosomes, confirming that kinetochores were still non-functional (Figure 9 and Figure S8). The inability of GFP-tailswap to load onto meiotic kinetochores suggests that the meiosis-specific CENH3 loading pathway is still functional even if we impose mitotic chromosome-like behaviour in meiotic cells by removing REC8 from centromeres.

### GFP-tailswap reloads onto mitotic kinetochores after meiosis

**Figure 10:**
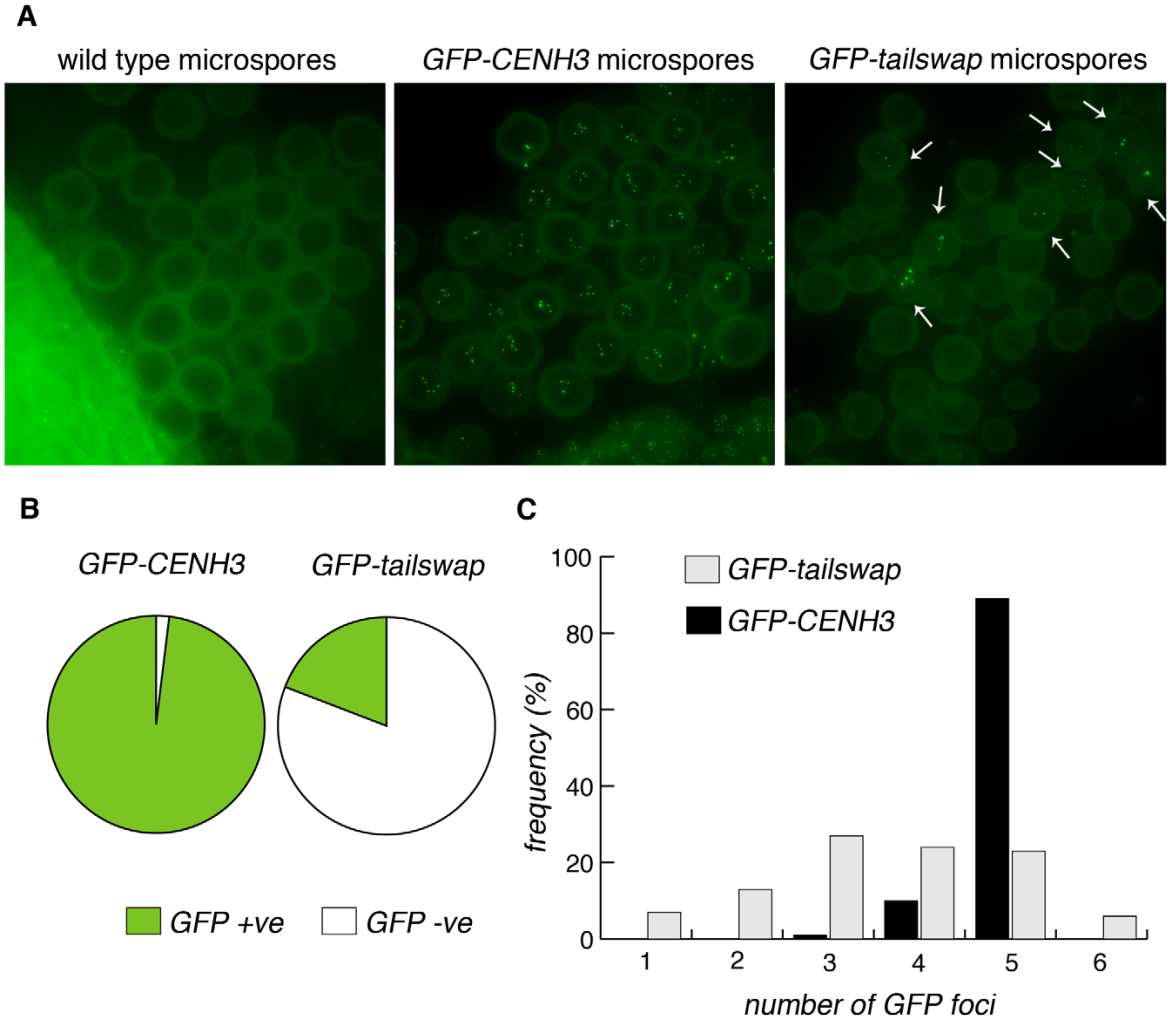
GFP-tailswap protein reloads onto centromeres after meiosis, when mitosis resumes. a) Microspores of wild type, *GFP-CENH3* and *GFP-tailswap*. Tetrad equivalent stages in *GFP-tailswap* do not show any GFP fluorescence at meiotic kinetochores (Figure S4). However, GFP-tailswap protein reloads onto kinetochores in a small fraction of microspores, when haploid mitotic divisions are expected to resume. b) Frequency of GFP-positive and -negative microspores from *GFP-CENH3* and *GFP-tailswap* plants. c) The number of GFP foci in each GFP-positive spore is shown.

Unlike animal gametes, the haploid cells produced by plant meiosis undergo mitotic divisions to generate mature gametes. Male meiosis in *GFP-tailswap* produces a few viable pollen grains, presumably in those rare cases where a complete haploid genome is clustered into a single microspore nucleus. These haploid microspores (and rarely, viable aneuploid microspores) must undergo mitotic divisions, so we asked whether the GFP-tailswap protein was recruited to kinetochores after meiosis, when mitosis resumes. Although a large majority of *GFP-tailswap* microspores (407/504 or 81%, n = 6 plants) are dead due to micronuclei formation and did not show GFP fluorescence, remaining microspores (97/504 or 19%) in the mutant showed GFP fluorescence at kinetochores at a level equivalent to wildtype (Figure 10A and 10B).

Some of the microspores that showed kinetochore fluorescence contained fewer than five GFP foci, and are unlikely to contain a full haploid genome (these microspores should lead to inviable pollen) (Figure 10C). Kinetochore localization of GFP-tailswap in haploid spores was brighter than that seen in early stages of meiosis (class II and class III). Furthermore, we did not observe GFP-tailswap at kinetochores from pachytene until telophase II of meiosis. We conclude that GFP-tailswap is loaded afresh onto mitotic kinetochores after meiosis and is not simply carried over from those meiocytes that showed faint localization. This result confirms the existence of two distinct kinetochore assembly pathways, for mitosis and meiosis respectively. It also raises the question of how the GFP-tailswap variant of CENH3 recognizes mitotic kinetochores after meiosis. If GFP-tailswap is truly removed from kinetochores in meiosis, there must be a targeting mechanism that does not require the prior presence of CENH3 in centromeric nucleosomes.

## Discussion

Centromeres are differentially configured during mitotic and meiotic cell divisions, resulting in separation of either sister chromatids or homologous chromosomes during anaphase. Segregation of sister chromatids to the same spindle pole in meiosis I depends on meiosis-specific proteins such as monopolin components and Moa1p, but may also involve specialized functions of constitutive kinetochore proteins. The essential kinetochore protein CENP-C recruits Moa1p in *S. pombe* [35]. In maize, MIS12 has a role in fusing kinetochores, facilitating mono-orientation during meiosis I [4]. In addition, linker histone H1 variants have a meiosis-specific role in plants and are present only at meiotic centromeres in lily [36]–[39]. CENH3 is essential in both mitosis and meiosis, but our viable yet sterile *A. thaliana* mutants have uncovered a meiosis-specific loading pathway for CENH3 that most likely depends on its fast evolving N-terminal tail domain. Male and female meiosis are differentially affected in *GFP-tailswap* plants, although the basis for this is unclear [14]. Sex specific meiotic chromosome segregation defects have also been observed in tobacco plants defective for linker histone H1 variants [39].

During DNA replication, CENH3 nucleosomes are randomly partitioned between replicated sister chromatids and voids created in chromatin are filled by fresh loading of free CENH3. The cell cycle loading time of CENH3, and the chaperones that direct it to kinetochores, can vary between organisms [16], [40], [41] [42]–[45]. Our results may indicate that CENH3 has meiosis-specific chaperones that cannot recruit the GFP-tailswap and GFP-maizetailswap variants. In *C. elegans*, CENH3/HCP-3 is also differentially recruited during mitosis and meiosis [46]. Furthermore, *C. elegans* CENH3 has a different distribution from outer kinetochore proteins in meiosis, and is dispensable for meiotic chromosome segregation [46], [47]. These properties are probably related to the holocentric nature of *C. elegans* chromosomes, whereas the meiosis-specific defect we have found confirms that CENH3 is essential for meiotic segregation of monocentric *A. thaliana* chromosomes.

Severe depletion of GFP-tailswap at meiotic kinetochores suggests that endogenous CENH3 undergoes a removal and reloading process in meiosis. If this is the case, reloading must be closely coupled to removal, as we never observed meiotic cells from *GFP-CENH3* plants that lacked kinetochore fluorescence. There are precedents for dynamic reorganization of CENH3 chromatin during particular developmental stages. In *C. elegans* meiosis, CENH3/HCP-3 is removed, and kinetochores are assembled *de novo* when mitosis resumes [46]. Parental CENH3 is also greatly depleted in the fertilized zygote of *A. thaliana*, and replaced by CENH3 synthesized from the zygotic genome [48]. In an alternative model to explain *GFP-tailswap* dysfunction, CENH3 at meiotic kinetochores might assume a different conformation that is destabilized by the combination of a bulky tag with the absence of the CENH3 N-terminal tail domain. Meiosis specific kinetochore architecture could involve an altered nucleosome structure (such as a conversion between octameric and tetrameric forms), or a change in the arrangement of CENH3 nucleosomes [49]–[51]. CENH3 levels can be regulated by proteolysis [52], and GFP-tailswap and GFP-maizetailswap proteins may be specifically degraded in meiotic cells (possibly because they are not loaded into centromeric chromatin). The fact that *A. thaliana* has a centromere DNA structure similar to most animals and plants favors the hypothesis that meiosis-specific CENH3 assembly will be a conserved phenomenon.

The involvement of the CENH3 N-terminal tail in meiosis-specific centromere assembly is intriguing because the tail is so fast-evolving, and because it is dispensable for accurate mitosis. This requirement is not absolute, as the H3.3 tail substitution in the absence of GFP is fertile – others have proposed that a GFP tag on CENH3 might disturb higher order chromatin structure [15]. The CENH3 tail (81 amino acids) is longer than the H3 tail (43 amino acids), so GFP is closer to the histone-fold domain in the GFP-tailswap protein. However, we do not think this is the sole cause of sterility in *GFP-tailswap*, because *GFP-maizetailswap* plants are even more sterile, and the maize CENH3 tail (61 amino acids) is longer than the H3 tail. The N-terminal tail of histone H3 can contain many functionally important post-translational modifications, and CENH3 can also be post-translationally modified [53], [54]. Meiosis-specific modifications of CENH3 seem unlikely to be significant, because the amino acid sequence of the tail changes so rapidly, and important modified residues would be expected to be conserved. The N-terminal tail may interact with as yet unidentified meiosis-specific histone chaperones, with other proteins important for mono-orientation, or even with centromere DNA directly [55]. If the latter is the case, this binding must be especially critical during meiosis.

The “centromere paradox” refers to the fact that centromere DNAs and CENH3 are remarkably fast evolving, despite their essential function [56]. It has been proposed that differences between the centromeres of two parents cause hybrid defects when they are crossed, leading to speciation [56]. Centromere differences could reduce the fitness of hybrid offspring by affecting either meiosis or mitosis. We previously reported that a cross between two phenotypically indistinguishable parents with CENH3 differences (*GFP-CENH3* and wild type) caused mitotic chromosome segregation errors in the fertilized zygote [14]. Now we have found that meiosis (particularly in the male) can be specifically affected by changes in CENH3, albeit in plants that contain only a single altered CENH3 protein. This result is analogous to the observation that a centromere DNA polymorphism in the monkeyflower *Mimulus guttatus* can cause male meiotic defects when it is homozygous [57]. We speculate that CENH3 interactions with centromere DNA may be altered by the *M. guttatus* centromere DNA polymorphism, and that *A. thaliana* GFP-tailswap protein may fail in meiosis because it cannot interact with centromere DNA appropriately. The existence of a meiosis-specific loading pathway for CENH3 further supports the concept that rapid evolution reduces hybrid fertility by weakening kinetochore function in meiosis.

## Materials and Methods

### Plant growth and materials

Plants were grown under a 16 hr light/8 hr dark regime at 20°C. *GFP tailswap* plants have been previously described [14], [13]. Plant transformations used the floral dip method. The *rec8* (L*er* accession) and *spo11-1* (Ws-0 accession) mutants have been described [3]. *rec8 spo11-1 cenh3-1* triple mutants expressing *GFP-tailswap* were generated by selfing *REC8/rec8 SPO11-1/spo11-1 CENH3/cenh3-1* plants carrying the *GFP tailswap* transgene. Primer sequences for genotyping are listed in Table S1.

### Plasmids and transgenes

The *GFP-maizetailswap* transgene fuses the *Zea mays* CENH3 N-terminal tail (amino acids 1–61) and the *A. thaliana* CENH3 histone fold domain (amino acids 82–179). *GFP-maizetailswap* was constructed by overlapping PCR and cloned as a *SalI-XbaI* fragment into the binary vector CP93 [13]. The *tailswap* transgene without an N-terminal GFP was constructed by overlapping PCR and cloned into CP93 from *SalI* to *XbaI*. Primer sequences for overlapping PCR are listed in Table S1.

### Differential interference contrast microscopy

Developmental analysis of unfertilized fixed ovules by differential interference contrast microscopy was performed as described [58].

### Meiotic chromosome spreads and FISH

Male meiotic spreads were prepared as described [59] except for a modification in the enzyme cocktail for tissue digestion, which contained 0.3% cellulose and 0.3% pectolyase in 10 mM citrate buffer (pH 4.5). FISH analysis on male meiotic chromosome spreads was performed as described [13]. The distance between centromeres at the metaphase I to anaphase I transition was measured using NIH Image J software.

### Immunolocalization analysis

Immunolocalization of alpha-tubulin in meiocytes and microspores was carried out as described [60]. The primary antibody was a mouse monoclonal anti-alpha-tubulin (Sigma T6199). The secondary antibody was a goat anti-mouse IgG (Sigma F0257). Images were captured with a Deltavision deconvolution microscope. Immunolocalization of GFP and MIS12 was performed as described previously [28]. We used an anti-GFP antibody from Acris (R1461P).

### GFP localization in meiocytes

To visualize GFP fluorescence in meiocytes, anthers were dissected from unfixed, fresh flower buds using insulin needles in a drop of staining solution (50% glycerol, 1% PBS and 1 µg/ml DAPI). After removing other floral tissues, anthers were fully submerged by fresh addition of 10–20 µl of staining solution onto the slide. A thin coverslip was placed on the slide and gently pressed with the plunger end of the insulin syringe until meiocytes were finely extruded out from the anther sacs. After sealing the coverslip with valap wax (vaseline∶lanolin∶paraffin wax in a 1∶1∶1 ratio), slides were imaged using a Deltavision deconvolution microscope. Images were captured at 60× magnification with an exposure time of 0.5 seconds. Z-stacks with a step size of 0.2 µM were captured and further transformed into two-dimensional (2D) flattened projections using SoftWoRx software (Applied Precision). TIF files were edited using Adobe Photoshop and Illustrator.

In live meiocytes stained with DAPI, it was difficult to accurately pinpoint the early stages of meiosis, especially the premeiotic and early prophase I stages. However, we could gauge the approximate stage by looking for certain landmark phenotypes. In the premeiotic stage, the chromosomes are highly decondensed and diffuse, and thus DAPI stains the whole nucleus. Therefore, premeiotic stage meiocytes were identified as being spherical or round in appearance. In premeiotic stage meiocytes, the G1 phase cells were identified as ones that showed round/spherical GFP fluorescence, whereas in S-G2 phase meiocytes, GFP signals are more elongated as a result of chromosome doubling. We never observed the clear separation of replicated sister kinetochores seen in mitotic G2 cells (paired GFP foci). As meiosis proceeds, chromosomes start to condense. When the round appearance of chromosome mass became more irregular, they were identified as leptotene stage meiocytes. During zygotene, pairing of homologous partners starts and hence there were 5–10 GFP signals corresponding to the centromeres. During pachytene, pairing is complete and thus we detected 5 GFP signals corresponding to 5 fused kinetochores. In pachytene, chromosomes threads are much compact than early stages. From metaphase I onwards, meiotic stages were distinguished by their chromosome segregation behaviour using DAPI staining.

### Meiocyte extraction

Male meiocytes were extracted with a microcapillary-based method as described previously [26].

## Supporting Information

Figure S1 Analysis of male and female sterility in *GFP-tailswap* plants. A. Pollen viability assayed by Alexander staining. Viable pollen stains red and dead pollen stains green. All pollen from wild type and *GFP-CENH3* plants were viable whereas 95% of pollen from *GFP-tailswap* were dead. B. Developmental analysis of female gametogenesis in *GFP-CENH3* and *GFP-tailswap* ovules. Around 30% of the developing ovules from GFP-tailswap plants either arrested at a single cell stage or did not have an embryo sac. mi- micronuclei, dg- degenerating, ap- antipodals, cc- central cell, ec- egg cell, sy- synergids, sa- single celled arrest.
(TIF)

Figure S2 Prophase I stages of meiosis I are normal in *GFP-tailswap* plants. (A,D) Pachytene stage showing normal condensation and pairing of homologous chromosomes. (B,E). Late diplotene stage showing chiasmata (arrows). (C,F). Diakinesis stage showing maximum condensation of bivalents. Scale bars −1 µm.
(TIF)

Figure S3 *GFP-maizetailswap* plants are sterile because of random chromosome segregation in meiosis. A. Vegetative phenotype of *GFP-maizetailswap* plants (arrows). Pollen from *GFP-maizetailswap* plants stains green and is thus inviable. Around 95% of the ovules did not contain an embryo sac (B) or arrested at the single celled stage (C) of gametogenesis. B. Male meiotic chromosome analysis in *GFP-maizetailswap* plants. A. Pachytene showing normal pairing. B. Late diplotene showing chiasmata (arrow) C. Diakinesis. D. Metaphase I. The bivalents were round rather than rhombus shaped. E, F. Anaphase I showing irregular chromosome segregation. Laggards can be seen in F. (arrow). G. Interkinesis showing irregular decondensation of chromosomes. H.-J. Metaphase II equivalent stages showing random distribution of chromosomes. In panel J, two of the chromosomes have separated their sister chromatids (arrow) whereas others have not and are randomly placed. K.,L. Anaphase II showing scattered segregation of sister chromatids. M,N. Early and late telophase II showing decondensation of chromatids at random locations. O. Polyad with multiple nuclei. P. Microspores with micronuclei. Scale bar −1 µm.
(TIF)

Figure S4 Anther squashes from *GFP-CENH3* and *GFP-tailswap* showing somatic cells (S) and meiocytes (M). Mitotic kinetochores show GFP fluorescence in both *GFP-CENH3* and *GFP-tailswap* somatic cells. GFP-CENH3 protein was present at kinetochores during all stages of meiosis. However, GFP-tailswap protein was undetectable in the majority of meiotic cells, beginning at pre-meiotic interphase until the completion of meiosis. White boxes in the merge panel indicate a single meiotic cell that is magnified in the rightmost panel.
(TIF)

Figure S5 Immunolocalization of GFP in *GFP-CENH3* (B,C) and *GFP-tailswap* (E-F) anther squashes (pachytene stage of meiosis I). GFP is seen at kinetochores of both somatic (arrows in B) and meiotic cells in *GFP-CENH3* anthers. In *GFP-tailswap*, only somatic cells (arrows in E) show GFP at kinetochores and meiotic cells are completely devoid of any GFP signal. Scale bar = 10 µm.
(TIF)

Figure S6 RT-PCR from *GFP-CENH3* and *GFP-tailswap* meiocytes and inflorescence cDNA. Inflorescence tissue contains somatic cells from anthers and ovules along with meiocytes. In all samples, we observed only the expected transcript size upon amplification, which was further confirmed by DNA sequencing. *GFP-CENH3* = 739 bp , *GFP-tailswap* = 625 bp. Primer sequences are listed in Table S1.
(TIF)

Figure S7 GFP-tailswap protein is not loaded onto centromeres even in presence of wild type CENH3. GFP-CENH3 is loaded effectively in the presence of wild type CENH3 in meiocytes (B,D) whereas GFP-tailswap is not detected at meiotic centromeres even in the presence of wild-type CENH3. The early microspores that are shed immediately after cytokinesis (J) also show no GFP signal .However, later stage microspores (L) show strong GFP signal as a result of fresh loading of GFP-tailswap once mitosis resumes. Panels E–L are derived from anthers from successive buds of a single inflorescence.
(TIF)

Figure S8 GFP-tailswap recruitment and meiotic chromosome segregation in *rec8 spo11-1 cenh3-1 GFP-tailswap* plants. A. Anther squashes from *rec8 spo11-1 cenh3-1 GFP-tailswap* plants. S- Somatic tissues. M- Meiocytes. GFP-tailswap protein can be detected in somatic cells of the anthers whereas it is not present in meiocytes at all stages of meiosis. However in a fraction of microspores that probably resume mitosis, GFP-tailswap protein is loaded back into centromeres. B. Male meiotic chromosome analysis in *rec8 spo11-1 cenh3-1 GFP-tailswap* plants. A.Interkinesis/dyad stage showing decondensation of 10 sister chromatids on either side of the organelle band in *rec8 spo11-1*. In *rec8 spo11-1 cenh3-1 GFP-tailswap* plants there is irregular partitioning of the chromosomes during meiosis I and II. A few chromosomes (especially the laggards that remain in the spindle midzone (arrows in C and E)) do not show any signs of decondensation during interkinesis.
(TIF)

Table S1 Primer sequences used in this study.
(DOC)
