# Supplementary figures and images for: Meiosis-Specific Loading of the Centromere-Specific Histone CENH3 in Arabidopsis thaliana
Source: PLoS Genet. 2011 Jun 9;7(6):e1002121. doi: 10.1371/journal.pgen.1002121 (PMC3111537; doi:10.1371/journal.pgen.1002121)

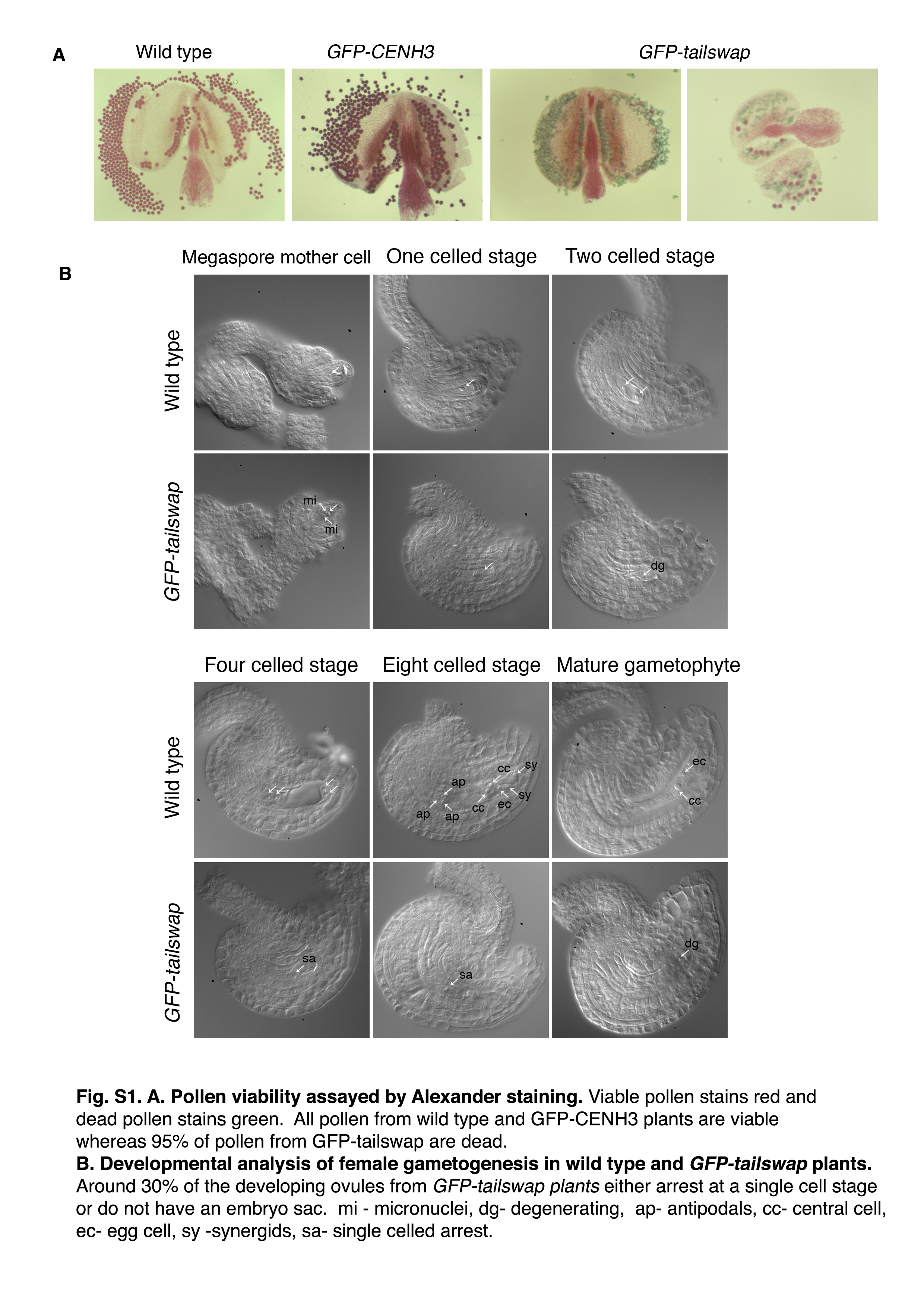

Supplement: Figure S1 — Analysis of male and female sterility in GFP-tailswap plants. A. Pollen viability assayed by Alexander staining. Viable pollen stains red and dead pollen stains green. All pollen from wild type and GFP-CENH3 plants were viable whereas 95% of pollen from GFP-tailswap were dead. B. Developmental analysis of female gametogenesis in GFP-CENH3 and GFP-tailswap ovules. Around 30% of the developing ovules from GFP-tailswap plants either arrested at a single cell stage or did not have an embryo sac. mi- micronuclei, dg- degenerating, ap- antipodals, cc- central cell, ec- egg cell, sy- synergids, sa- single celled arrest. (TIF) [file pgen.1002121.s001.tif]

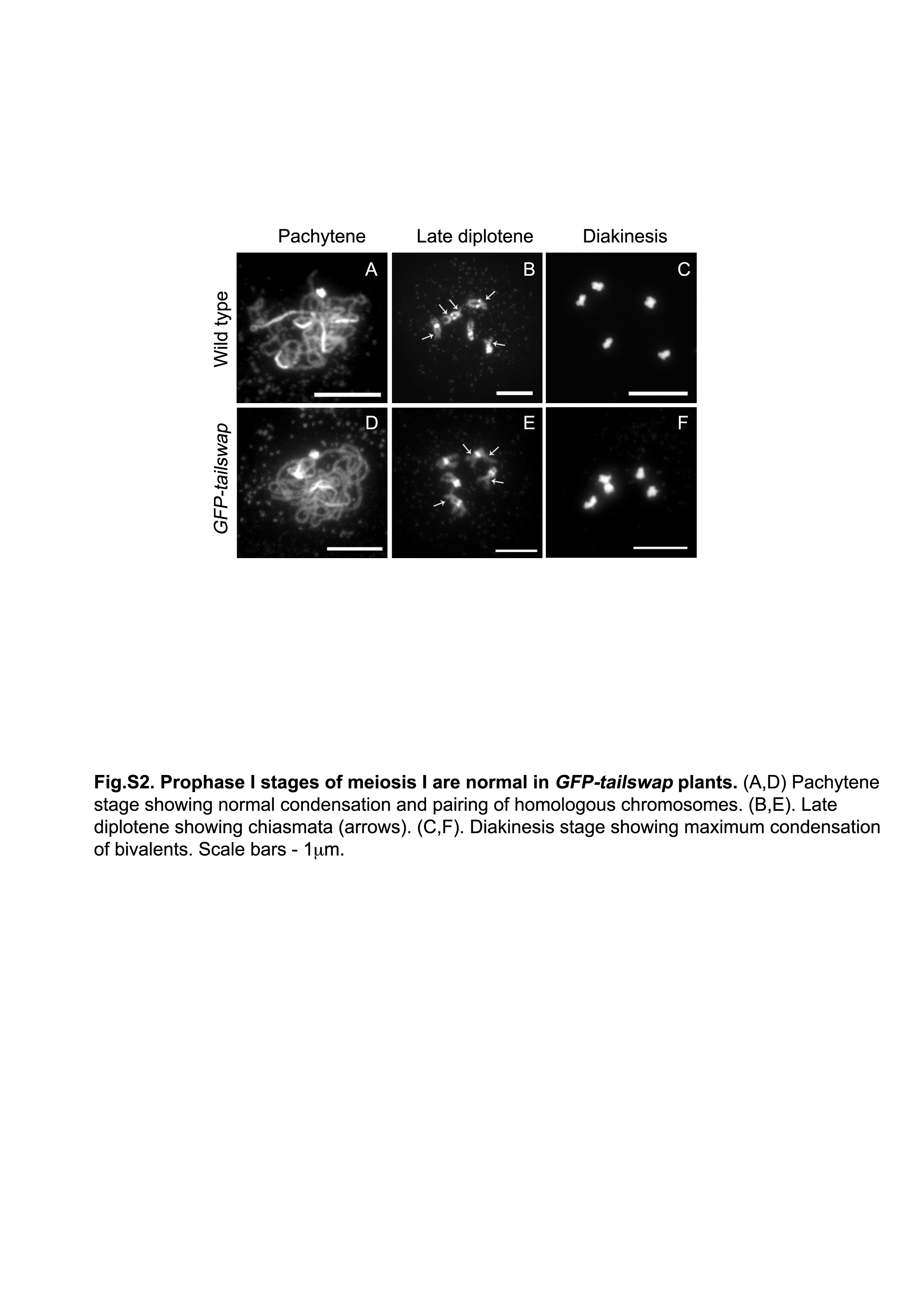

Supplement: Figure S2 — Prophase I stages of meiosis I are normal in GFP-tailswap plants. (A,D) Pachytene stage showing normal condensation and pairing of homologous chromosomes. (B,E). Late diplotene stage showing chiasmata (arrows). (C,F). Diakinesis stage showing maximum condensation of bivalents. Scale bars −1 µm. (TIF) [file pgen.1002121.s002.tif]

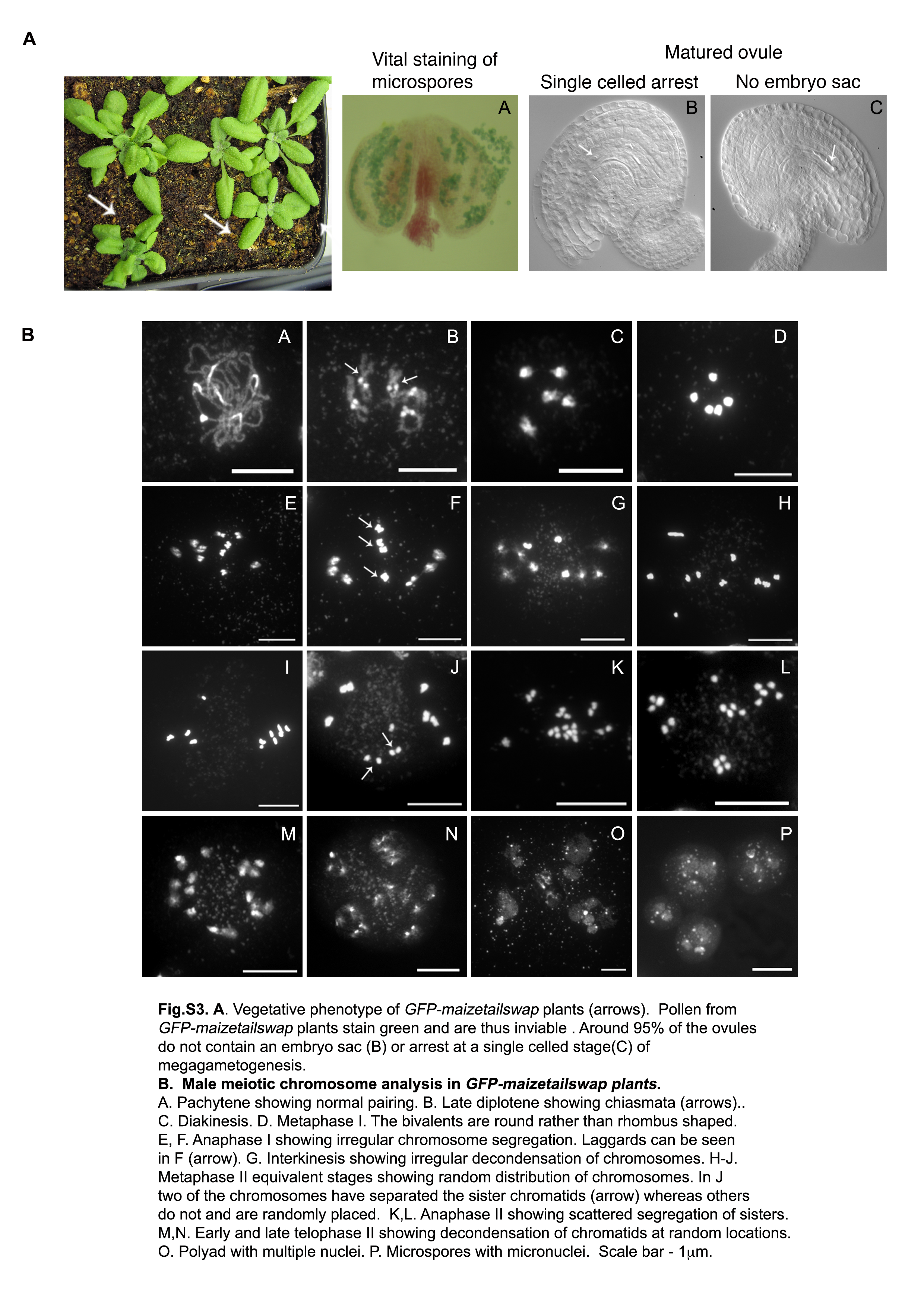

Supplement: Figure S3 — GFP-maizetailswap plants are sterile because of random chromosome segregation in meiosis. A. Vegetative phenotype of GFP-maizetailswap plants (arrows). Pollen from GFP-maizetailswap plants stains green and is thus inviable. Around 95% of the ovules did not contain an embryo sac (B) or arrested at the single celled stage (C) of gametogenesis. B. Male meiotic chromosome analysis in GFP-maizetailswap plants. A. Pachytene showing normal pairing. B. Late diplotene showing chiasmata (arrow) C. Diakinesis. D. Metaphase I. The bivalents were round rather than rhombus shaped. E, F. Anaphase I showing irregular chromosome segregation. Laggards can be seen in F. (arrow). G. Interkinesis showing irregular decondensation of chromosomes. H.-J. Metaphase II equivalent stages showing random distribution of chromosomes. In panel J, two of the chromosomes have separated their sister chromatids (arrow) whereas others have not and are randomly placed. K.,L. Anaphase II showing scattered segregation of sister chromatids. M,N. Early and late telophase II showing decondensation of chromatids at random locations. O. Polyad with multiple nuclei. P. Microspores with micronuclei. Scale bar −1 µm. (TIF) [file pgen.1002121.s003.tif]

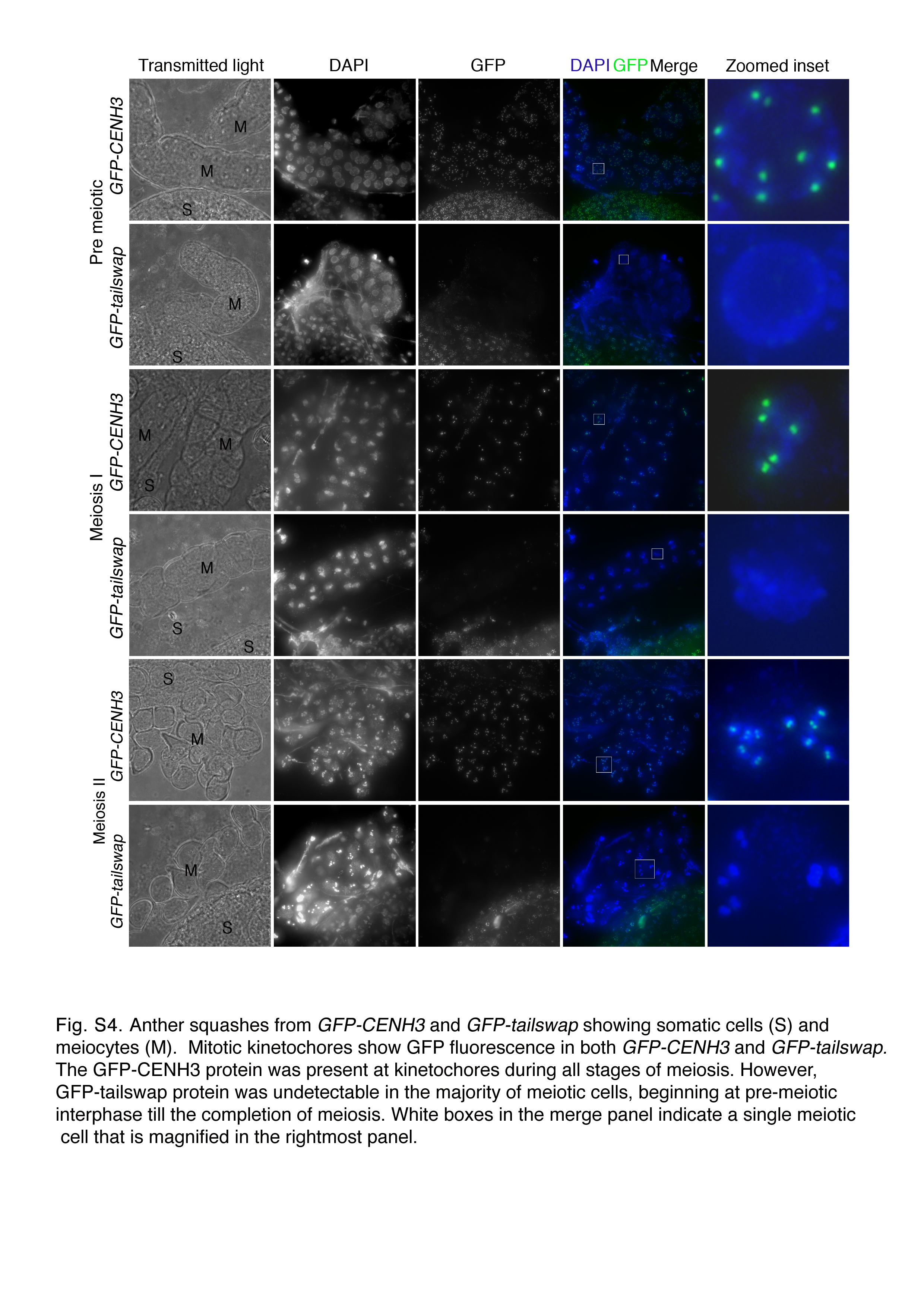

Supplement: Figure S4 — Anther squashes from GFP-CENH3 and GFP-tailswap showing somatic cells (S) and meiocytes (M). Mitotic kinetochores show GFP fluorescence in both GFP-CENH3 and GFP-tailswap somatic cells. GFP-CENH3 protein was present at kinetochores during all stages of meiosis. However, GFP-tailswap protein was undetectable in the majority of meiotic cells, beginning at pre-meiotic interphase until the completion of meiosis. White boxes in the merge panel indicate a single meiotic cell that is magnified in the rightmost panel. (TIF) [file pgen.1002121.s004.tif]

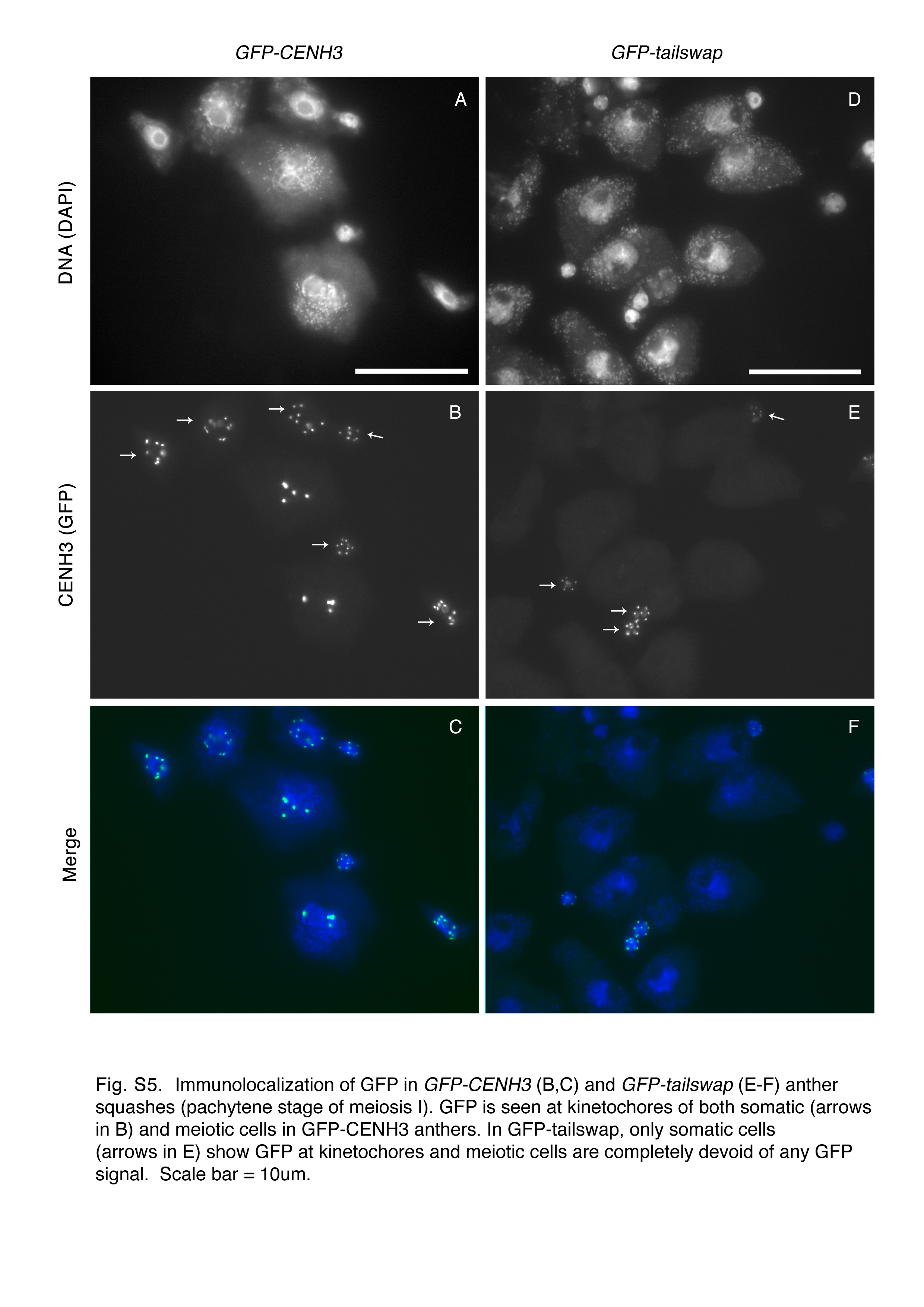

Supplement: Figure S5 — Immunolocalization of GFP in GFP-CENH3 (B,C) and GFP-tailswap (E-F) anther squashes (pachytene stage of meiosis I). GFP is seen at kinetochores of both somatic (arrows in B) and meiotic cells in GFP-CENH3 anthers. In GFP-tailswap, only somatic cells (arrows in E) show GFP at kinetochores and meiotic cells are completely devoid of any GFP signal. Scale bar = 10 µm. (TIF) [file pgen.1002121.s005.tif]

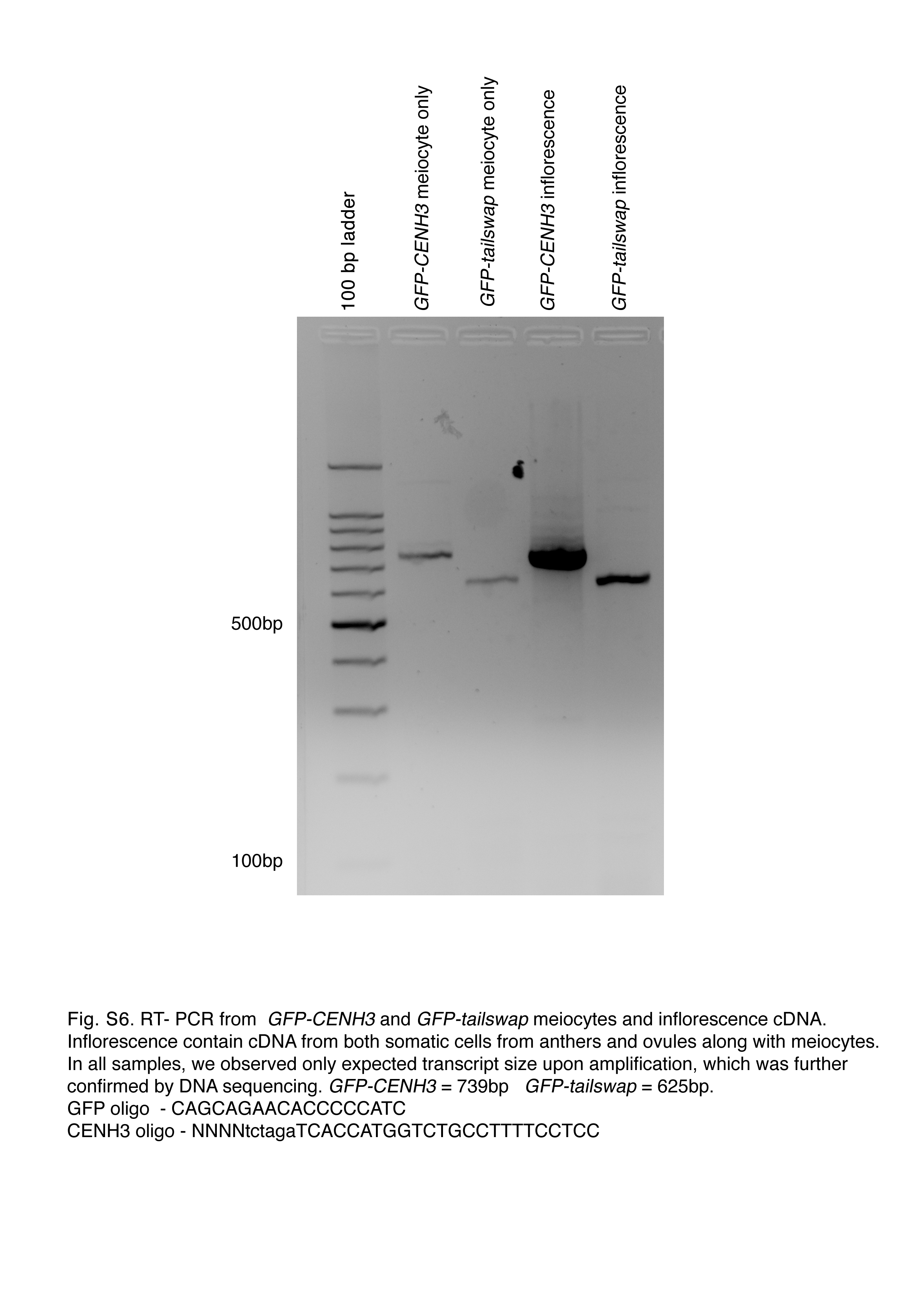

Supplement: Figure S6 — RT-PCR from GFP-CENH3 and GFP-tailswap meiocytes and inflorescence cDNA. Inflorescence tissue contains somatic cells from anthers and ovules along with meiocytes. In all samples, we observed only the expected transcript size upon amplification, which was further confirmed by DNA sequencing. GFP-CENH3 = 739 bp , GFP-tailswap = 625 bp. Primer sequences are listed in Table S1. (TIF) [file pgen.1002121.s006.tif]

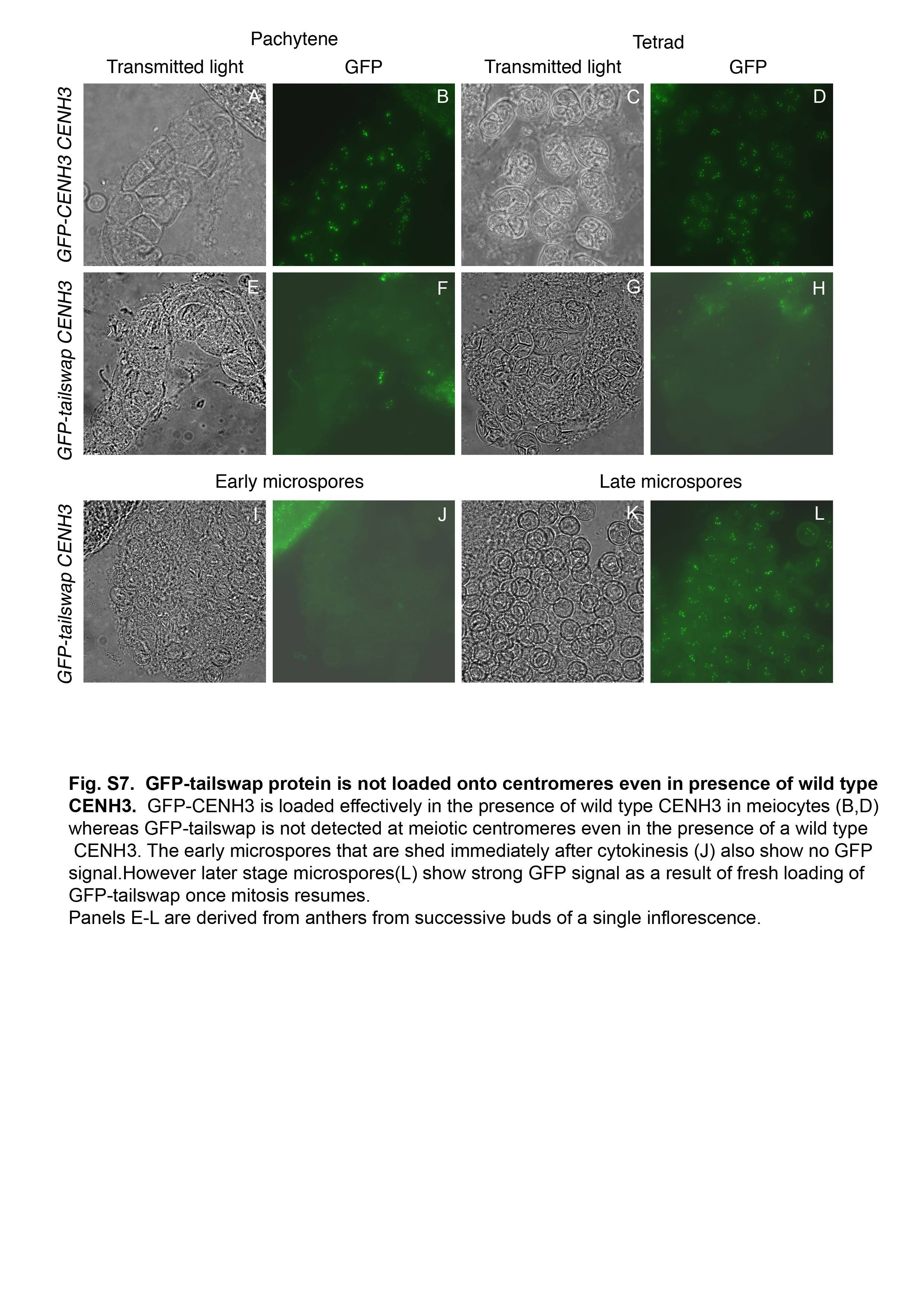

Supplement: Figure S7 — GFP-tailswap protein is not loaded onto centromeres even in presence of wild type CENH3. GFP-CENH3 is loaded effectively in the presence of wild type CENH3 in meiocytes (B,D) whereas GFP-tailswap is not detected at meiotic centromeres even in the presence of wild-type CENH3. The early microspores that are shed immediately after cytokinesis (J) also show no GFP signal .However, later stage microspores (L) show strong GFP signal as a result of fresh loading of GFP-tailswap once mitosis resumes. Panels E–L are derived from anthers from successive buds of a single inflorescence. (TIF) [file pgen.1002121.s007.tif]

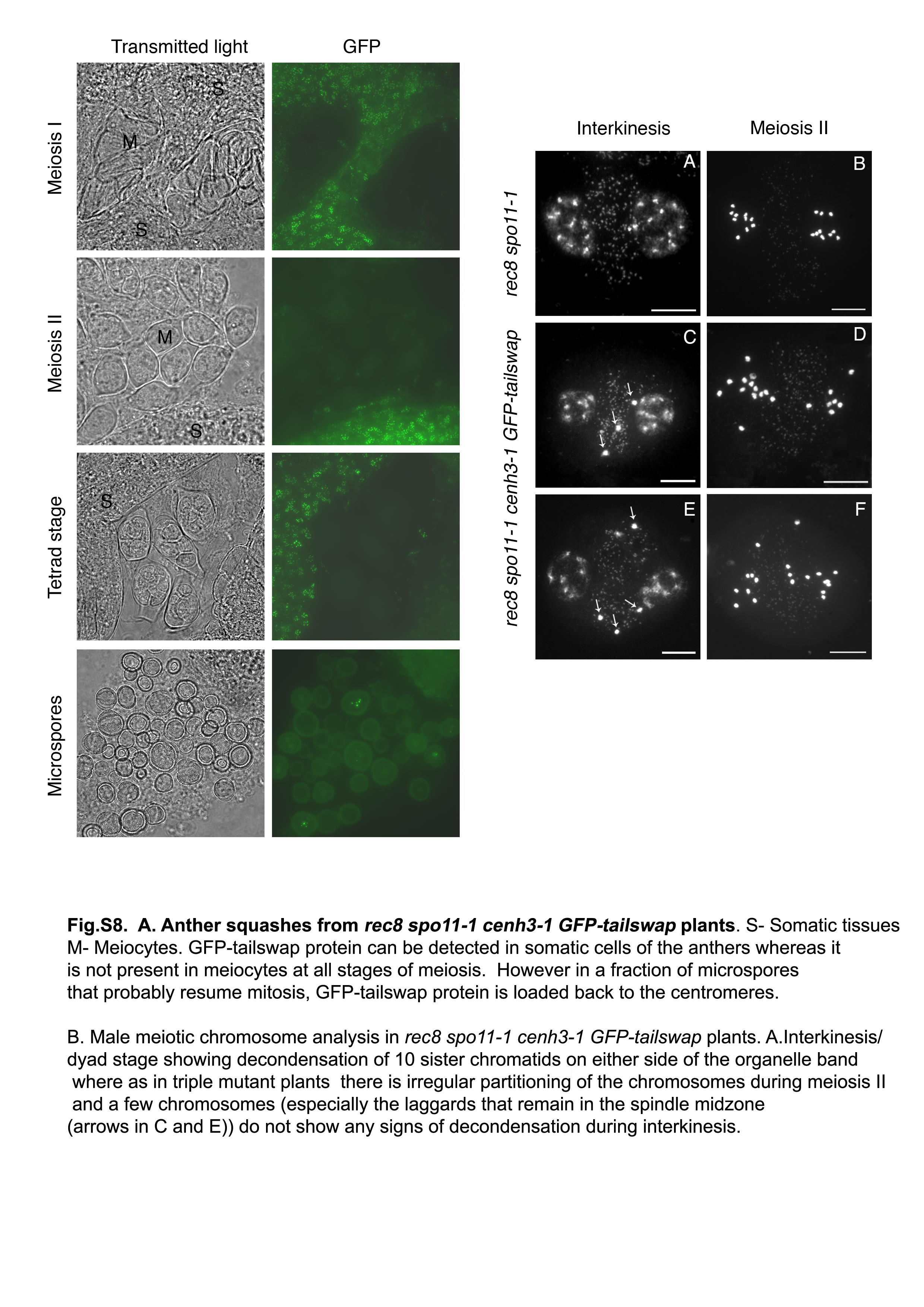

Supplement: Figure S8 — GFP-tailswap recruitment and meiotic chromosome segregation in rec8 spo11-1 cenh3-1 GFP-tailswap plants. A. Anther squashes from rec8 spo11-1 cenh3-1 GFP-tailswap plants. S- Somatic tissues. M- Meiocytes. GFP-tailswap protein can be detected in somatic cells of the anthers whereas it is not present in meiocytes at all stages of meiosis. However in a fraction of microspores that probably resume mitosis, GFP-tailswap protein is loaded back into centromeres. B. Male meiotic chromosome analysis in rec8 spo11-1 cenh3-1 GFP-tailswap plants. A.Interkinesis/dyad stage showing decondensation of 10 sister chromatids on either side of the organelle band in rec8 spo11-1. In rec8 spo11-1 cenh3-1 GFP-tailswap plants there is irregular partitioning of the chromosomes during meiosis I and II. A few chromosomes (especially the laggards that remain in the spindle midzone (arrows in C and E)) do not show any signs of decondensation during interkinesis. (TIF) [file pgen.1002121.s008.tif]
