# Supplementary material for: Meiosis-Specific Loading of the Centromere-Specific Histone CENH3 in Arabidopsis thaliana
Source: PLoS Genet. 2011 Jun 9;7(6):e1002121. doi: 10.1371/journal.pgen.1002121 (PMC3111537; doi:10.1371/journal.pgen.1002121)
Supplement: Table S1 — Primer sequences used in this study. (DOC) [file pgen.1002121.s009.doc]

| **Sl. No** | **Name** | **Oligo sequence (5’ -3’)** | **Gene** | **Remarks** |
| --- | --- | --- | --- | --- |
| 1 | CP 498 | GGTGCGATTTCTCCAGCAGTAAAAATC | *CENH3*  *(HTR12)* | **Genotyping *cenh3-1* locus**  Digestion of PCR product with EcoRV *cenh3-1* allele is uncut (215 bp), *CENH3* allele gets cleaved to give (191bp + 24bp) |
| 2 | CP 499 | CTGAGAAGATGAAGCACCGGCGATAT | *CENH3*  *(HTR12)* |
| 3 | CP 638 | CTGAAGCTGAACCTTCGTCTCG | Cytokinin oxidase 5 (*CKX 5*) | **Genotyping *GFP-tailswap* insertion**  CP 638 + CP 639 = WT locus specific amplification  CP 639 + CP 367 – T-DNA insertion specific amplification |
| 4 | ÇP 639 | CACATACTCGCTACTGGTCAGAGAATC | Cytokinin oxidase 5 |
| 5 | CP 367 | AATCCAGATCCCCCGAATTA | T -DNA Left border |
| 6 | CP 646 | TGATCTTCGCGTGCAATGTAGC | *AtREC8* | **Genotyping *REC8* locus**  CP 646 + CP 647 - WT locus specific amplification  CP 646 + CP 648 – Transposon insertion specific amplification |
| 7 | CP 647 | GCCGATGCGAACTTCAATGG | *AtREC8* |
| 8 | CP 648 | ATACGATAACGGTCGGTAC | AcTransposon |
| 9 | CP 643 | ACTGGGATTCGTCTTGGACA | T -DNA Left border | **Genotyping *SPO11* locus**  CP 644 + CP 645 - WT locus specific amplification  CP 643 + CP 645 - T-DNA insertion specific amplification |
| 10 | CP 644 | TTTGGAGATCTTCCTTCAGCC | *AtSPO11* |
| 11 | CP 645 | ACGTATCGGGCCTAAATTCC | *AtSPO11* |
| 12 | CP 384 | NNNNGTCGACATGGCTCGAACCAAGCACCA | *ZmCENH3*  (Maize) | **To construct *GFP-Maize tailswap* chimeric DNA**  CP 384+CP 572 - Fragment 1  CP 571+CP 375 - Fragment 2  Fragment 1 and 2 were gel purified and fused by overlapping PCR using oligos CP384 + CP375 |
| 13 | CP 571 | GCCACCGCTGGAGGCCAGGAACCGTTG |  |
| 14 | CP 572 | CAACGGTTCCTGGCCTCCAGCGGTGGC |  |
| 15 | CP 375 | NNNNTCTAGATCACCATGGTCTGCCTTTTCCTCC | *CENH3*  *(HTR12)* |
| 16 | CP 404 | CAGCAGAACACCCCCATC | *GFP* | **RT-PCR and sequencing of cDNA from *GFP-CENH3* and *GFP-tailswap* meiocytes**  CP 404 + CP 375 |

**Supplementary Table 1 : Primers used in this study**
